# Dually Acting Ligands Targeting Serotonin Receptors: Implications in CNS Disorders

**DOI:** 10.1021/acs.jmedchem.5c03094

**Published:** 2026-04-27

**Authors:** Wojciech Trybała, Katarzyna Grychowska, Natalia Malikowska-Racia, Agnieszka Nikiforuk, Piotr Popik, Paweł Zajdel

**Affiliations:** † Chair of Bioorganic Chemistry, 49573 Jagiellonian University Medical College, 9 Medyczna Street, 30-688 Kraków, Poland; ‡ Doctoral School of Medical and Health Sciences, 49573 Jagiellonian University Medical College, 16 Łazarza Street, 31-530 Kraków, Poland; § Maj Institute of Pharmacology, Polish Academy of Sciences, 12 Smętna Str., 31-324 Kraków, Poland

## Abstract

The serotonergic system remains a critical focus of neuropsychopharmacology due to its widespread influence on mood, cognition, and behavior. Despite the clinical success of selective serotonin reuptake inhibitors (SSRIs), their long-term efficacy is limited by receptor heterogeneity, desensitization, and compensatory adaptations. Recent advances suggest that ligands simultaneously modulating two serotonin (5-HT) receptor subtypes may offer superior therapeutic outcomes. This perspective summarizes progress in developing such dually acting compounds for CNS disorders, including Alzheimer’s and Parkinson’s disease, schizophrenia, and mood disorders. Clinically relevant examples include flibanserin (5-HT_1A_ receptor agonist/5-HT_2A_ receptor antagonist), pimavanserin (5-HT_2A_/5-HT_2C_ receptors inverse agonist), and eltoprazine (5-HT_1A_/5-HT_1B_ receptors partial agonist), alongside experimental 5-HT_2A_/5-HT_6_, 5-HT_3_/5-HT_6_ or TAAR1/5-HT_2C_ receptors ligands. Integrating structure–activity insights and clinical findings, we discuss challenges of rational dual modulation. Advances in biased signaling, targeting distinctive conformational states, and optopharmacology utilizing photochromic ligands may further enable the design of innovative dually acting agents with improved efficacy and safety profiles.

## Graphical abstract

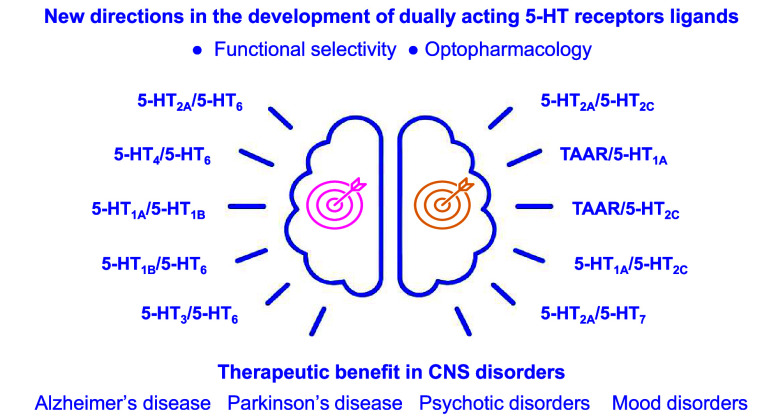

## 1 Statement of Significance

Dual serotonergic modulation offers a mechanistic way to address limitations of single-target CNS therapies. This perspective synthesizes clinical, pharmacological, and SAR evidence on approved and investigational dual-acting ligands to delineate when engaging two 5HT receptors confers therapeutic benefit. By including receptor state selectivity, biased signaling, and emerging photochromic paradigm, we illustrate how innovative dual mechanisms can expand therapeutic options and guide strategy in CNS drug discovery.

## 2 Introduction

Serotonin (5-hydroxytryptamine, 5-HT) is a neurotransmitter crucial for the regulation of both basal brain functions, which maintain vital life processes (e.g., respiration, blood pressure, motor control, balance) and higher brain functions relating to intellectual activities and consciousness.^1^ 5-HT is particularly well-known as a neurotransmitter but also acts as a neurotrophic factor during neurodevelopment.^2^

Serotonin signaling in the brain is tightly regulated at release sites at the presynaptic neurons by the serotonin transporter (SERT).^3^ Once released, 5-HT exerts its effects through a diverse family of 5-HT receptors, which are distributed throughout the brain and peripheral nervous system. These receptors are classified into seven families (5-HT_1_–5-HT_7_) based on their molecular structures and pharmacological functions. With the exception of the serotonin type 3 (5-HT_3_) receptor, which is a ligand-gated ion channel, all of them are G protein-coupled receptors (GPCRs), enabling complex intracellular signaling cascades.^4,5^ Excitatory signaling is primarily driven by receptors coupled to Gq/11 (e.g., 5-HT_2A_, 5-HT_2C_) and Gs/o (e.g., 5-HT_4_, 5-HT_6_, 5-HT_7_) proteins.^6^ Activation of Gq/11-coupled subtypes stimulates phospholipase C, while Gs-coupled subtypes enhance adenyl cyclase activity and cyclic adenosine monophosphate (cAMP) production to enhance cortical glutamate tone and synaptic plasticity. Conversely, inhibitory modulation is mediated by the Gi/o-coupled family, most notably the 5-HT_1_ receptors (5-HT_1A_, 5-HT_1B_, 5-HT_1D_, 5-HT_1E_, 5-HT_1F_) and 5-HT_5_ subclasses. These receptors inhibit adenylyl cyclase and hyperpolarize neurons and suppression of the neurotransmitter release. Since 5-HT receptors play a pivotal, yet diverse, role in balancing excitatory and inhibitory signals in the brain, they are promising targets for treating various disorders of the central nervous system (CNS).^7,8^

Apart from the SERT and above heterogeneous family of receptors, serotonergic homeostasis is auxiliary controlled by the low-affinity transports. Specifically, organic cation transporter 3 (OCT3) and the plasma membrane monoamine transporter (PMAT) act as an auxiliary mechanism controlling 5-HT.^9^ These transporters, expressed in cortical and limbic regions and located in the postsynaptic neurons, contribute to clearance of serotonin when the high-affinity SERT is saturated or inhibited.^10^

Historically, CNS drug discovery relied on phenotypic screening, where compounds were selected based on observable behavioral effects without detailed knowledge of their molecular mechanisms. This approach frequently led to discovery of agents with a complex polypharmacological profile, an outcome that was typically characterized and understood retrospectively rather than being a planned design objective. Advances in molecular biology and structural pharmacology shifted the field toward target-based drug design, focusing on well-defined molecular structures such as receptors and transporters. In light of the long-recognized stagnation in psychiatric drug approvals, such rational, mechanism-driven design is increasingly vital, illustrating how targeted drug development can replace serendipity. While target-based paradigm facilitated the development of even better follower drugs, in many cases, targeting a single receptor subtype with selective ligands has not achieved satisfactory therapeutic outcomes. The complexity of serotonergic signaling and its extensive cross-talk with other neurotransmitter systems often limits treatment efficacy and elevates the risk of adverse effects. Consequently, there is growing interest in developing dually acting compounds that can simultaneously modulate two receptor targets to overcome these therapeutic shortcomings and enhance clinical effectiveness.

The clinical use of selective serotonin reuptake inhibitors (SSRIs) illustrates both the promise and limitations of single-mechanism therapies. SSRIs are the most commonly prescribed medications for major depressive disorders, but their therapeutic effects typically appear after several weeks. To accelerate therapeutic effects, efforts have turned to dual-action strategies, deliberately combining blockade of SERT with the modulation of 5-HT_1A_ or 5-HT_2C_ receptors.^11^ Vilazodone exemplifies this approach, exhibiting SERT inhibitory activity with partial 5-HT_1A_ receptor agonism (Figure 1). This dual mechanism is thought to enhance anxiolytic effects and accelerate antidepressant response by alleviating the inhibitory feedback that constrains 5-HT release.^12^ Similar mechanistic insight guided the target-based design of vortioxetine, which displays a unique pharmacological profile among a class of SERT inhibitors (Figure 1). Through the structure–activity relationship (SAR) optimization, vortioxetine was engineered to combine inhibitory effect at SERT with multimodal activity, specifically a full 5-HT_1A_ receptor agonism and antagonism at 5-HT_3_ receptor.^13^ This receptor-targeting diversity is believed—based on preclinical studies—to contribute to a shortened latency in the onset of therapeutic effect. Of note, vortioxetine’s procognitive properties, demonstrated in clinical trials, distinguish it from other antidepressants and highlight its potential utility in addressing cognitive deficits associated with depression.^14^ These examples underpin a growing class of multimodal CNS agents designed to optimize therapeutic outcomes by engaging multiple serotonergic targets.

**Figure 1:**
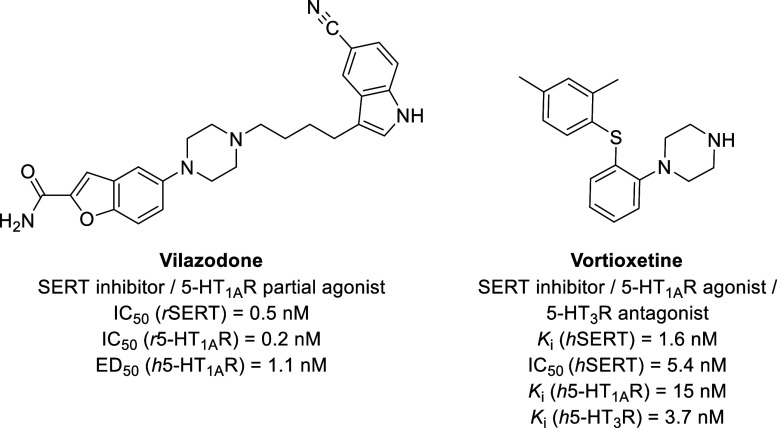
Vilazodone, a dually acting antidepressant drug, which behaves as a SERT inhibitor and a partial agonist at 5-HT_1A_ receptors.^15^ Vortioxetine, a multimodal antidepressant drug, which combines inhibitory activity at SERT with 5-HT_1A_ receptor agonism and antagonism at 5-HT_3_ receptor.^13^ Vilazodone: *r*SERT: [^3^H]-5-HT reuptake inhibition assay (rat synaptosomes); 5-HT_1A_ receptor: [^3^H]-8-OH-DPAT binding (rat hippocampus membranes) and [^35^S]-GTPγS functional assay (CHO cells).^15^ Vortioxetine: *h*SERT: [^3^H]-*S*-citalopram binding (Peakr293 cells) and [^3^H]-5-HT uptake inhibition (CHO cells); *h*5-HT_1A_ receptor: [^3^H]-8-OH-DPAT binding (CHO cells); *h*5-HT_3A_ receptor: [^3^H]-granisetron binding (HEK-293 cells).^13^

Beyond mood disorders, dual serotonergic modulation has been successfully explored in other therapeutic areas. Flibanserin’s modulation of serotonergic pathways was linked to mood regulation, motivation, and reward. It primarily behaves as a dually acting 5-HT_1A_ receptor agonist and 5-HT_2A_ receptor antagonist, reducing 5-HT inhibitory control over noradrenergic and dopaminergic signaling and enhancing the mesocorticolimbic pathway (Figure 2). It also exhibits additional affinities, within a similar order of magnitude to its 5-HT_1A_ activity, including weaker antagonistic actions at 5-HT_2C_ and 5-HT_2B_ receptors, and a less characterized interaction with dopamine type 4 (D_4_) receptor. However, the clinical relevance of these secondary interactions is considered minor.^16^ This mechanism was found particularly effective in restoring hypoactive sexual desire. Although originally developed as an antidepressant, flibanserin was approved for the treatment of hypoactive sexual desire disorder (HSDD) in premenopausal women.^17^

**Figure 2:**
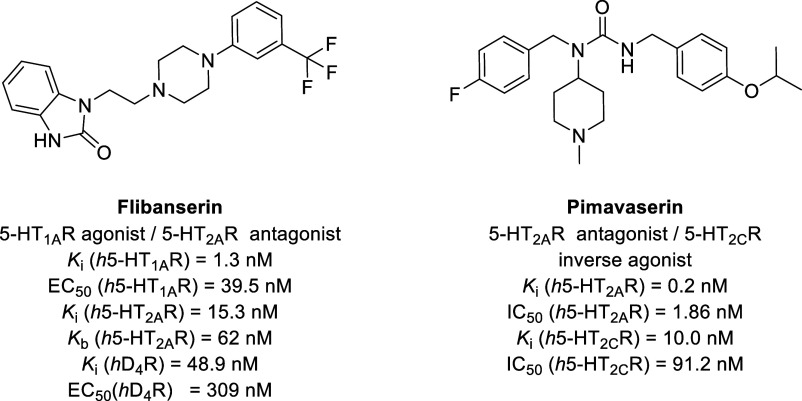
Flibanserin, originally developed as an antidepressant and later repurposed for HSDD in premenopausal women, exerts its effects through agonist activity at 5-HT_1A_ receptor and antagonist activity at 5-HT_2A_ receptor.^16,20^ Pimavanserin is a dually acting compound which behaves as inverse agonist at 5-HT_2A_ receptor-operated Gα_i_1 signaling, neutral antagonist at 5-HT_2A_ receptor-operated the Gq/11 signaling and inverse agonist at 5-HT_2C_ receptor, indicated for the treatment of Parkinson’s disease psychosis (PDP).^18,21^ Flibanserin: *h*5-HT_1A_ receptor: [^3^H]-8-OH-DPAT binding (CHO cells) and cAMP functional agonism (CHO cells); *h*5-HT_2A_ receptor: [^3^H]-ketanserin binding (CHO cells) and *h*5-HT_2A_ receptor antagonism (CHO cells); *h*D_4_ receptor: [^3^H]-spiperone binding (CHO cells) and [^35^S]-GTPγS functional assay (CHO cells).^16,20,22^ Pimavanserin: *h*5-HT_2A_ receptor: [^3^H]-ketanserin binding (HEK-293T) and R-SAT functional assay (NIH-3T3 cells); *h*5-HT_2C_ receptor: [^3^H]-mesulergine binding (HEK-293T) and R-SAT functional assay (NIH-3T3).^21^

Soon thereafter, the Food and Drug Administration (FDA) approved pimavanserin. This compound displays distinctive dual activity at 5-HT_2A_ and 5-HT_2C_ receptors. It behaves as inverse agonist at the 5-HT_2A_ receptor operated Gα_i_1 signaling pathway which has been implicated as a noncanonical effector contributing to hallucinogenic responses and neutral antagonism at the canonical Gq/11 pathway. It simultaneously acts as inverse agonism at the 5-HT_2C_ receptor.^18^

Unlike classical antipsychotics, pimavanserin does not antagonize dopamine type 2 (D_2_) receptor and therefore avoids worsening extrapyramidal motor dysfunction in patients with Parkinson’s disease. Its specificity for serotonergic receptors underscores its unique therapeutic profile, offering benefits in neuropsychiatric conditions without the motor side effects associated with dopaminergic blockade (Figure 2).^19^

Evidence for the utility of dual serotonergic ligands also extends to the field of migraine therapy. Although not designed for CNS disorders, triptans, such as sumatriptan, were the first highly selective, dually acting 5-HT receptor ligands successfully used in the clinic since the 1990s (Figure 3). The initial design was to elaborate compounds selective for the 5-HT_1_-like receptor family, which were implicated in migraine. In the 1980s, the classification of the 5-HT_1_ receptor subclass was still evolving, so that characterization of sumatriptan’s binding profile was rather deconvolution than a preplanned scenario. A majority of triptans activate 5-HT_1B_ and 5-HT_1D_ receptors to reverse arterial dilation and reduce release of proinflammatory neuropeptides, such as calcitonin gene-related peptide (CGRP) thereby aborting migraine attacks.^23^ Recent advancement in the field of triptans involved introduction of naratriptan, which additionally activates 5-HT_1F_ receptor. This broader receptor engagement further suppresses central pain signaling and lowers cardiovascular risks associated with earlier-generation triptans. Importantly, the role of 5-HT_1F_ receptors was highlighted with the approval of lasmiditan, the first drug to act selectively at this site. Unlike triptans, lasmiditan does not constrict blood vessels but alleviates migraine pain by modulating and calming trigeminal nerve activity, offering an option for patients with cardiovascular risk.^24^

**Figure 3:**
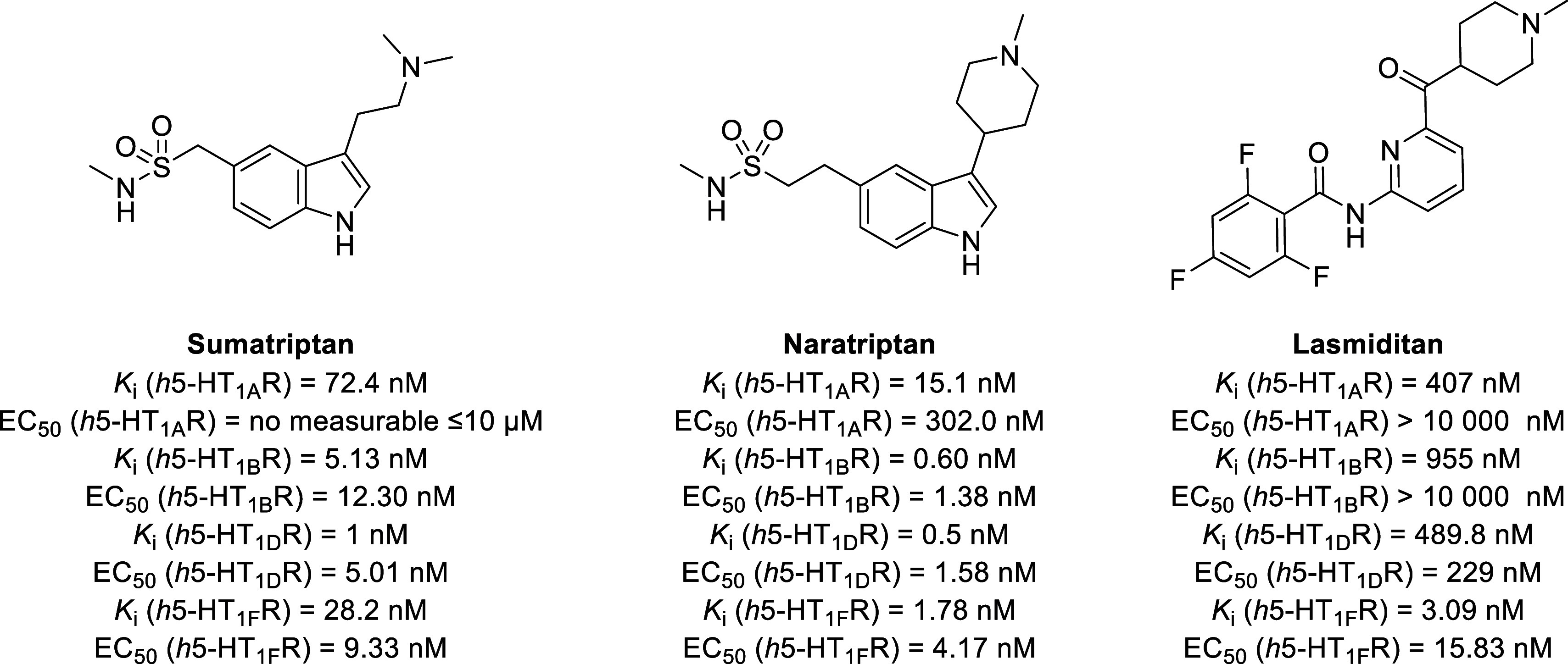
Sumatriptan, the first approved triptan, selectively activates 5-HT_1B_ and 5-HT_1D_ receptors to alleviate acute migraine attacks. Naratriptan, a newer agent, additionally targets 5-HT_1F_ receptor, which helps to suppress central pain transmission. Many triptans also bind at 5-HT_1A_ receptors, though most exhibit little or only weak functional 5-HT_1A_ agonism. Lasmiditan, a later development, is a selective 5-HT_1F_ receptor agonist, offering a safer alternative for patients with cardiovascular risk. For all compounds: radioligand displacement binding (*h*5-HT_1A_ receptor [^3^H]-8-OH-DPAT, *h*5-HT_1B_/*h*5-HT_1D_ receptors [^3^H]-5-CT, *h*5-HT_1F_ receptor [^3^H]-LSD; CHO-K1 cells expressing respective receptors); [^35^S]-GTPγS functional assay in CHO-K1 cells membranes expressing respective receptor.^25^

Thus, converging lines of evidence suggest that interest in exclusive and dually acting serotonergic compounds is steadily increasing. This developmental approach offers the benefits of multimodal 5-HT receptors targeting, while selectivity toward specific targets avoids adverse effects and unexpected interactions. In this review, we discuss how a similar approach guides strategies of SAR in drug development, shaping new research directions across various CNS disorders.

The selection of compounds discussed in this review was primarily guided by their developmental status and pharmacological relevance within the serotonergic system.

Compounds combining SERT with additional activities at 5-HT receptors,^26–29^ at dopamine D_2_ receptors, sigma receptors, and acetyl cholinesterase were widely described in the literature and are the subject of comprehensive reviews on multitarget compounds, such as those by Spinks D. and Spinks G.^11^ and Ghafir El Idrissi I. et al.^30^ In contrast, other published studies have described additional multitarget concepts (e.g., targeting 5-HT_2C_/5-HT_6_ ^31^ or 5-HT_1A_/5-HT_7_ ^32–34^ receptors), but these are largely supported only by *in vitro* data. Accordingly, our review focus is more specific.

In this perspective, we specifically included ligands demonstrating dual activity exclusively at serotonin receptor subtypes but not at SERT, OCT3, and PMAT proteins. Inclusion criteria further mandated either progression to clinical trials or comprehensive evaluation in advanced preclinical models. Data sources comprised peer-reviewed publications and registered clinical trial databases. The resulting selection aims to provide a representative overview of the most promising dually acting serotonergic ligands within the current drug discovery landscape.

## 3 Dual Serotonergic Modulation in Neurodegenerative Disorders

The pathophysiology of neurodegenerative disorders involves complex, multifactorial mechanisms characterized by progressive neuron loss, synaptic dysfunction, and disruption of neuronal networks. These conditions pose significant challenges for early diagnosis, effective therapeutic intervention, and management of cognitive and functional decline. Despite extensive research efforts, current treatments primarily offer symptomatic relief, with limited capacity to modify disease progression. Major neurodegenerative diseases, including Alzheimer’s disease (AD), Parkinson’s disease (PD), Huntington’s disease (HD), and amyotrophic lateral sclerosis (ALS), exhibit distinct molecular and cellular pathologies that complicate the development of targeted, disease-modifying therapies. Addressing the inherent complexities of these disorders remains a critical goal in neurobiology, aiming to develop disease-modifying strategies and ultimately enhance patient’s health outcomes.

### 3.1 Alzheimer Disease

AD is characterized by progressive decline in memory functions. Despite advances in understanding their molecular underpinnings, the pathogenic mechanisms remain incompletely understood, underscoring the urgent need for innovative therapeutic strategies to halt or reverse neuronal degeneration and preserve neurological function.

#### 3.1.1 Dually acting 5-HT_2A_/5-HT_6_ receptors antagonists: preclinical rationale and clinical data

Early research identified the potential of 5-HT_6_ receptor as a therapeutic target for cognitive deficits, particularly in neurodegenerative and psychiatric disorders; for review, see refs 35–37 . For example, SB-258585,^38^ SB-399885,^39^ and intepirdine^40^ which behave as 5-HT_6_ receptor inverse agonists and likewise CPPQ (compound 42 in ref 41) and IIQ^42^ (compound 25 in ref 43) which behave as neutral antagonists at 5-HT_6_ receptor-operated Gs signaling alleviated phencyclidine (PCP)-induced cognitive deficits in the novel object recognition (NOR) test in rats.

This potential is rooted in its predominant localization in brain regions linked to memory and cognition, including the hippocampus, striatum, nucleus accumbens, and prefrontal cortex. The 5-HT_6_ receptor belongs to the family of GPCRs, which are positively coupled with adenylyl cyclase through Gs protein. Additionally, 5-HT_6_ receptor engages the extracellular signal-regulated kinase (ERK)­1/2 pathway via the Src family tyrosine kinase Fyn,^44^ as well as cyclin-dependent kinase type 5 (Cdk 5)^45^ and the mechanistic target of the rapamycin (mTOR) pathway. As revealed by works of Dr. Philippe Marin’ team, the 5-HT_6_ receptor-operated Cdk5 pathway plays a critical role in neurodevelopment, including neuronal migration and differentiation.^45^ Beyond its classical coupling to Gs protein, the 5-HT_6_ receptor also activates mTOR signaling, which has been shown to impair cognitive functions in schizophrenia,^46^ preclinical models of adolescent cannabis abusers.^47^ These observations indicate that inhibiting the 5-HT6 receptor–mTOR signaling pathway represents a promising therapeutic approach for mitigating cognitive deficits across diverse neurodegenerative and neuropsychiatric conditions.

In parallel, the 5-HT_2A_ receptor represents another serotonergic target implicated in psychotic mechanisms, whose modulation may further enhance the therapeutic efficacy of 5-HT_6_ receptor antagonists. These receptors are widely expressed in the brain, including dopaminergic neurons in the substantia nigra and ventral tegmental area, where their activation enhances striatal dopamine release, underlying psychotic effects. Conversely, blockade of 5-HT_2A_ receptors prevents abnormal excitation of dopaminergic neurons, underlying the efficacy of many atypical antipsychotics. 5-HT_2A_ receptors are also abundant on cortical GABAergic interneurons and glutamatergic pyramidal neurons. Their blockade enhances excitatory cortical feedback to the dorsal raphe nucleus (DRN), thereby increasing 5-HT release and restoring cortical neural balance.^48,49^

In addition to the use in PD, pimavanserin is currently under clinical investigation for the treatment of general dementia-related psychosis (HARMONY, No. NCT03325556),^50^ including AD. Because psychosis is a common comorbidity in AD, selective 5-HT_2A_ receptor inverse agonists may be a new treatment alternative, with a more favorable safety profile in elderly patients.

Collectively, 5-HT_2A_ antagonism or inverse agonism, in addition to antipsychotic effect, may augment the procognitive efficacy of 5-HT_6_ receptor antagonists (see above^35^). Thus, dually acting 5-HT_2A_/5-HT_6_ receptors antagonists represented a promising treatment strategy for AD.

Intepirdine (RVT-101) is an arylsulfonyl quinoline derivative that was originally developed as a selective 5-HT_6_ receptor antagonist (Figure 4). Although explicit medicinal chemistry literature detailing the stepwise SAR that led to the identification of intepirdine is limited, patent WO 03/080580 A2 describes the structural modifications and optimization applied to this group of compounds.

**Figure 4:**
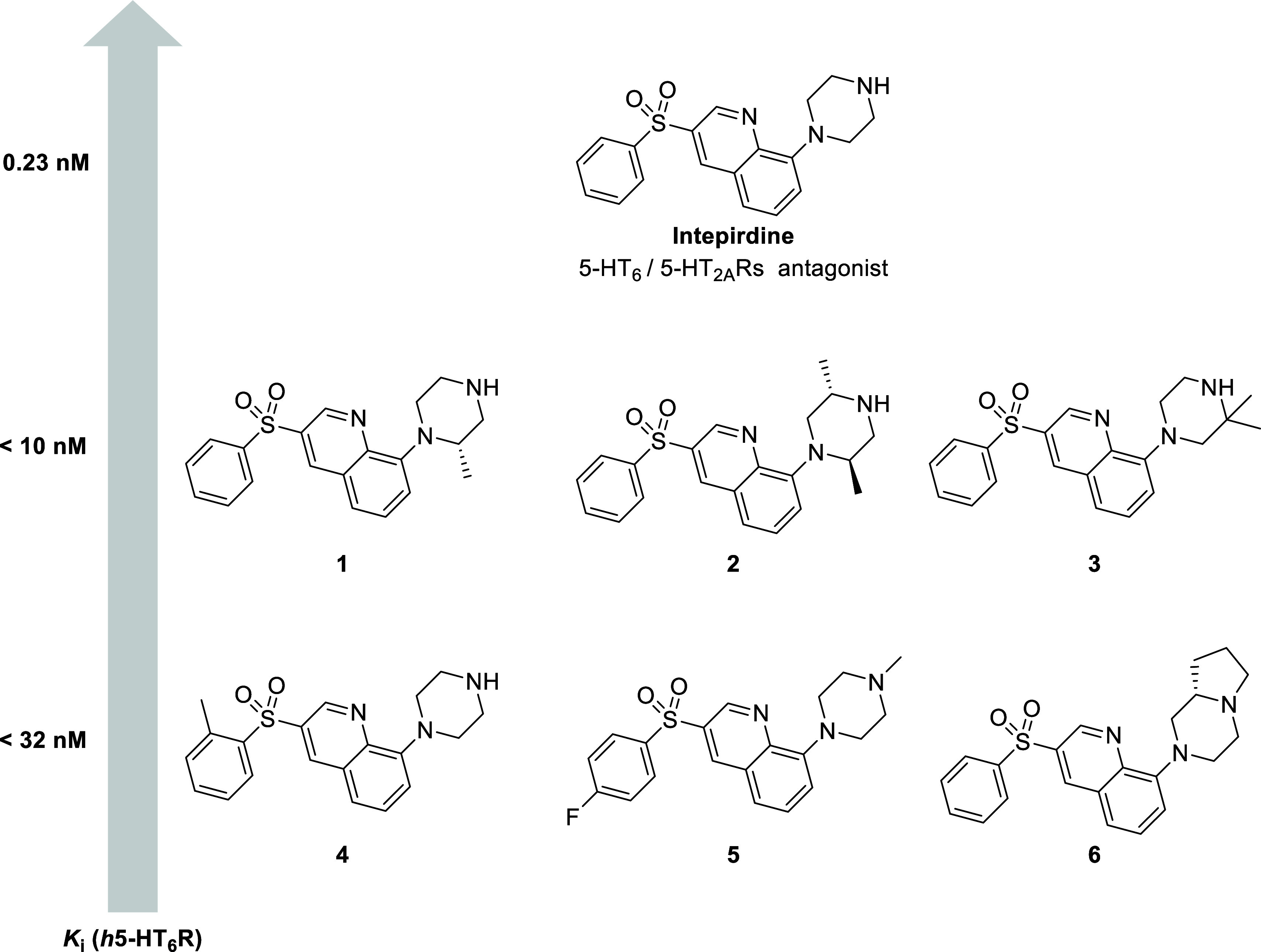
Structural modifications of 3-phenylsulfonyl-8-piperazine-1-yl quinoline leading to the identification of intepirdine. *K*_i_ values refer to the *h*5-HT_6_ receptor, determined by [^3^H]-LSD (HeLa cells), assay per WO 03/080580;^51^ methods as in WO 98/27081).^52^

Structural diversification comprised the introduction of various substituents in the arylsulfonyl fragment, as well as modification of the piperazine ring with alkyl groups. Among the synthesized analogs, derivatives bearing a methyl group at the 2-position or a dimethyl substitution at the 3-position of the piperazine ring exhibited a high affinity for the 5-HT_6_ receptor, with *K*_i_ values below 10 nM.

Intepirdine has been further characterized as an inverse agonist at 5-HT_6_ receptor, as demonstrated in NG108-15 transiently expressing 5-HT_6_ receptor, a cellular model in which the receptor displays high constitutive activity.^53^ Furthermore, it was classified as an antagonist of 5-HT_2A_ receptor (*K*_i_ = 10 nM).^54^

Although a clear synergistic contribution of 5-HT_2A_/5-HT_6_ receptors modulation has not been demonstrated in the context of intepirdine in AD, the compound has been extensively investigated to enhance cognitive function in neurodegenerative disorders such as AD and dementia with Lewy bodies (DLB). In animal studies, intepirdine has been shown to improve cholinergic neurotransmission, promote neural plasticity, and enhance cognitive function.^54^ Furthermore, it has been reported to potentiate the effects of donepezil on cholinergic signaling, highlighting its potential role in combination therapy aimed at mitigating cognitive decline.

While intepirdine was one of the most extensively evaluated 5-HT_6_ receptor antagonists, it ultimately failed in phase III clinical trials when tested as an add-on therapy to donepezil. The reason for this fiasco remains unclear; however, it has been attributed to the differences in the design of phase III and phase II clinical trials including variation in dosage regimens.^55^

Building on previous research, Staroń et al. described development of dual antagonists targeting 5-HT_6_ and 5-HT_2A_ receptors among tryptamine analogs. These compounds were identified through a virtual screening approach, followed by iterative hit-to-lead optimization.^56^ Out of the initially 14 identified hits, seven demonstrated moderate to high affinity for 5-HT_6_ receptor, and one of them—tryptamine derivative **7**—was selected for further development due to its favorable binding profile (Figure 5).

**Figure 5:**
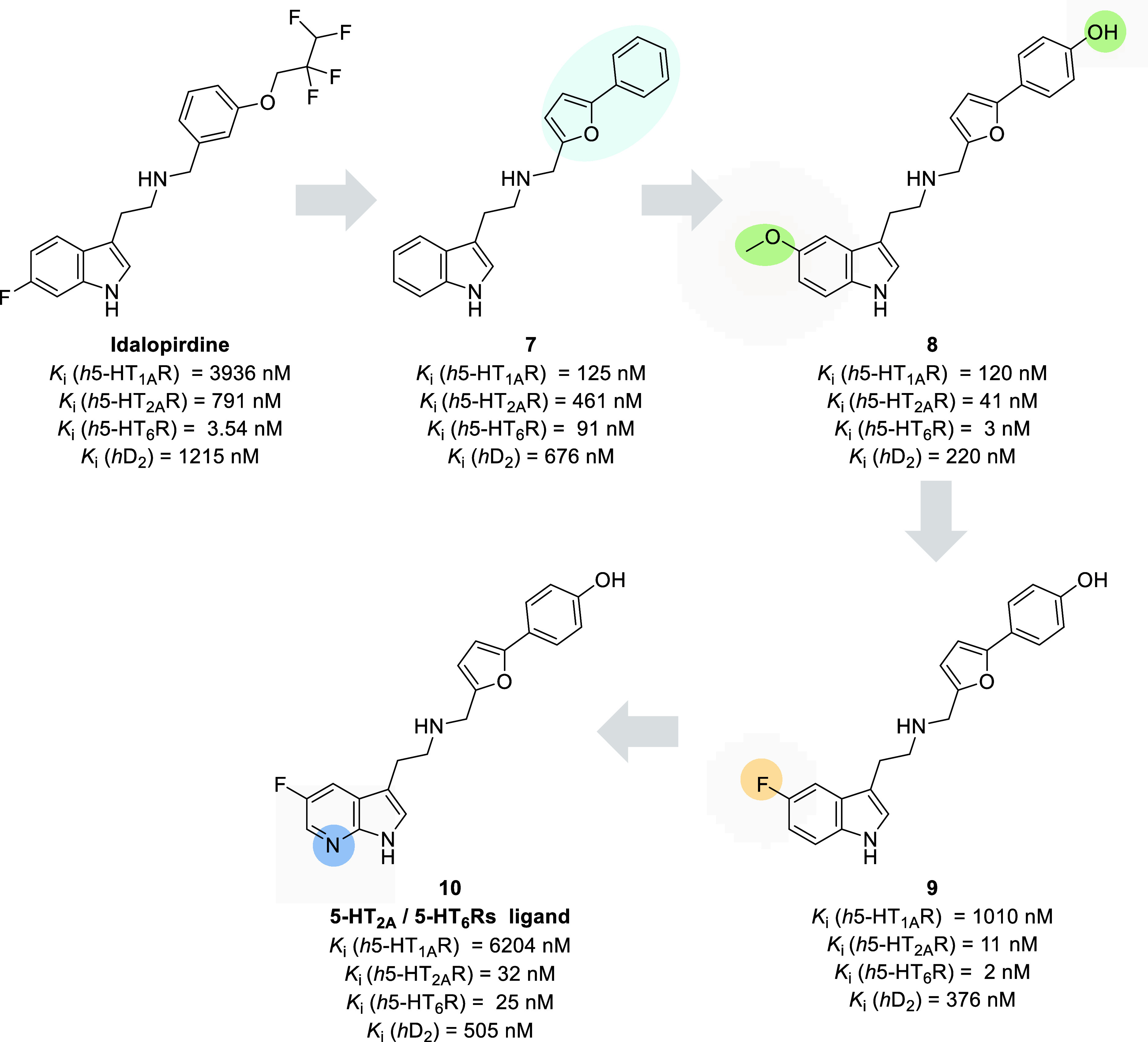
Structures of idalopirdine and novel tryptamine derivatives developed as dually acting 5-HT_2A_/5-HT_6_ receptors ligands. [^3^H]-8-OH-DPAT for *h*5-HT_1A_ receptor, [^3^H]-ketanserin for *h*5-HT_2A_ receptor, [^3^H]-LSD for *h*5-HT_6_ receptor, and [^3^H]-raclopride for *h*D_2_ receptor radioligand binding (in HEK293 cells expressing the respective human receptor).^56^

SAR analysis of novel tryptamine derivatives targeting 5-HT_6_ and 5-HT_2A_ receptors highlighted several critical features guiding ligand optimization. Among heterocyclic variants, furyl-containing compounds consistently demonstrated superior affinity for 5-HT_6_ receptor compared to thiophene and pyrrole analogs. Substituents on the indole ring substantially influenced receptor binding: a methoxy group at C5, as in compound **8**, yielded nanomolar affinity for both 5-HT_6_ (*K*_i_ = 3 nM) and 5-HT_2A_ receptors (*K*_i_ = 41 nM), while its substitution with fluorine (compound **9**) preserved affinity for 5-HT_6_ receptor and 4-fold enhanced affinity for 5-HT_2A_ receptor. Importantly, the presence of a *para*-hydroxyl group on the phenyl moiety was associated with a significant increase in the affinity for 5-HT_6_ receptor, consistent with the trends observed across the series. Structural modifications such as replacement of the indole nitrogen with oxygen resulted in dramatic loss of activity, whereas introducing a nitrogen at the C7 position (compound **10**) produced a dual ligand, which behaved as a 5-HT_6_ receptor antagonist in the Gs pathway, with improved receptor selectivity and metabolic stability. Compound **10** was brain-penetrant (brain/plasma ≈0.7) and reversed PCP-induced cognitive impairment in the NOR test in rats at effective doses of 3 and 10 mg/kg (*po*, in male Sprague–Dawley rats). These insights emphasize the potential of rationally designed dual 5-HT_6_/5-HT_2A_ receptors antagonists as candidates for treating cognitive deficits in neuropsychiatric disorders.

Further mechanistic and translational studies are required to disentangle the contributions of specific 5-HT_2A_ receptor and 5-HT_6_ receptor signaling pathways (including receptor-state-dependent and biased signaling) and to verify their clinical relevance in neurodegeneration using standardized cognitive paradigms.

#### 3.1.2 Dually acting 5-HT_4_ receptor partial agonists/5-HT_6_ receptor antagonists: effects on cognition

The 5-HT_4_ receptor is a Gs-coupled serotonin receptor enriched in the hippocampus and cortex; its activation elevates cAMP-PKA/EPAC signaling, enhances acetylcholine release, and facilitates hippocampal synaptic plasticity, thereby counteracting cholinergic deficits in AD.^57,58^ In parallel, 5-HT_4_ agonism promotes nonamyloidogenic amyloid precursor protein (APP) processing via α-secretase, increasing sAPPα and reducing amyloid-β (Aβ) generation—supporting disease-modifying potential.^58^ Convergent preclinical data show robust procognitive effects of selective 5-HT_4_ receptor agonists (e.g., RS-67333, BIMU-8, SL65.0155, PRX-03140) across learning and memory paradigms.^57,59^ Quiedeville et al.^60^ showed that both the 5-HT_4_ receptor partial agonist RS-67333 and the 5-HT_6_ receptor antagonist SB-271046 improved the recognition index in the object recognition test in mice. The authors suggested that these effects may be mediated by enhanced glutamatergic and/or cholinergic neurotransmission, as 5-HT_4_ and 5-HT_6_ receptor blockade increases the release of acetylcholine or both acetylcholine and glutamate, respectively.^60^

Importantly, the selective activation of 5-HT_4_ receptor signaling has demonstrated translational relevance in humans. For example, administration of a single 1 mg dose of the highly selective 5-HT_4_ receptor agonist prucalopride significantly improved performance across multiple learning and memory tasks in healthy volunteers. Moreover, short-term dosing was associated with increased activation of memory-related brain regions, accompanied by measurable behavioral improvements.^61^ In addition, repeated dosing of prucalopride (1 mg/day) for 6 days increased neural activation in memory-associated regions (the hippocampus and right angular gyrus) during memory tasks with simultaneous improvement in hippocampal-dependent memory task performance.^62^ Consequently, the strategy of integrating 5-HT_4_ receptor agonistic activity with 5-HT_6_ receptor antagonism within a single molecule represents a novel approach providing both symptomatic relief and disease-modifying effects in AD. Such an approach was applied by the team of Patrick Dallemagne and Christophe Rochais, who merged the structural framework of RS67333 with the arylsulfonamide fragment characteristic of 5-HT_6_ receptor antagonists (Figure 6).^63^

**Figure 6:**
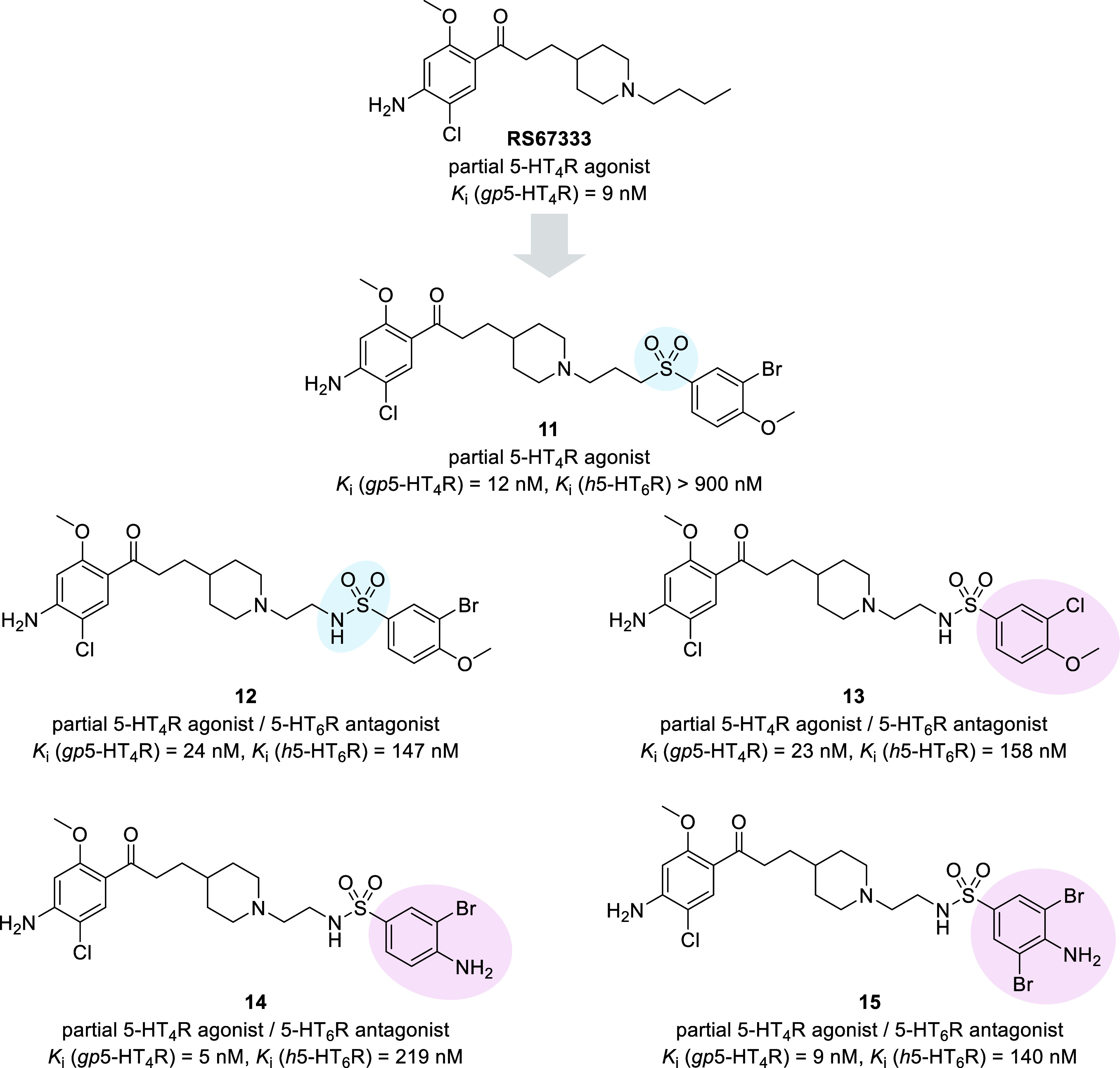
Structures of 5-HT_4_ receptor agonist RS67333 and dually acting compounds with affinity for 5-HT_4_ and 5-HT_6_ receptors designed as a new strategy for the treatment of AD. Radioligand binding: [^3^H]-GR113808 for *gp*5-HT_4_ receptor (guinea-pig striatal membranes); [^3^H]-LSD for *h*5-HT_6_ receptor (HEK-293 cells).^63^

The structural modifications comprised adjusting the distance between the arylsulfonylamide group and the piperidine nitrogen atom, replacing the sulfonamide group with a sulfone moiety and introducing various substituents within the arylsulfonyl fragment.

All compounds with the two-carbon linker between the basic nitrogen and the arylsulfonylamide group displayed high-to-moderate affinity for 5-HT_4_ receptor irrespective of their substituents on the phenyl ring (*K*_i_ = 2–50 nM). Among them, derivatives with either a *para* methoxy or an amino group on the phenyl ring and a second substituent in *ortho* position exhibited higher affinities than reference compound RS67333 (*K*_i_ = 9 nM).

Concerning 5-HT_6_ receptor, four compounds (**12**, **13**, **14**, and **15**) showed submicromolar affinities (*K*_i_ = 140–219 nM). All of these derivatives contained a *para* amino or a methoxyphenyl group in the arylsulfonamide fragment. The presence of a halogen atom on the arylsulfonamide ring seemed to be essential for 5-HT_6_ receptor activity. It is noteworthy that replacement of the sulfonamide group with sulfone was highly unfavorable for the affinity for 5-HT_6_ receptor (33% inhibition at 1 μM).

Further evaluation of the selected compound **14** revealed that it exhibits almost the same affinity for both 5-HT_6_ receptor and 5-HT_2B_ receptor (39% and 49% at 10^–8^ M, respectively). Moreover, it demonstrates affinity for the 5-HT_2A_ receptor (*K*_i_ = 330 nM) and the 5-HT_2C_ receptor (*K*_i_ = 160 nM). Compound **14** revealed antiamnesic activity at a dose of 1 mg/kg against scopolamine-induced impairment in the spontaneous alternation test in the mouse.

These observations suggest that the therapeutic approach combining 5-HT_4_ receptor agonism and 5-HT_6_ receptor antagonism offers a coherent and potentially synergistic mechanism to enhance cognition. By simultaneously boosting cholinergic and glutamatergic neurotransmission and mitigating pathological mTOR signaling, this dual approach provides a strong rationale for targeted intervention.

### 3.2 Parkinson’s Disease

PD is a progressive neurodegenerative disorder primarily characterized by motor symptoms such as tremors, rigidity, and bradykinesia. In addition to these motor features, many PD patients also experience cognitive decline, affecting memory, attention, and executive functions. The mainstay of motor symptom management involves the administration of levodopa (l-DOPA), typically combined with peripheral DOPA decarboxylase inhibitors and other agents aimed at enhancing dopaminergic transmission. While l-DOPA provides effective symptomatic relief during the early stages of the disease, long-term therapy is often hindered by notable challenges. These include the development of motor complications such as dyskinesias, fluctuations between on and off periods, and “wearing-off” phenomena. Furthermore, chronic treatment can lead to nonmotor adverse effects, including psychosis and autonomic dysfunction, which significantly impact patients’ quality of life. Addressing these therapeutic limitations remains a major focus of ongoing research in PD management.

#### 3.2.1 Dually Acting 5-HT_1A_/5-HT_1B_ Receptors Partial Agonists: Impact on Motor Control

Current treatments for PD primarily aim to enhance dopaminergic transmission. However, to mitigate the motor complications associated with long-term l-DOPA therapy, serotonergic agents offer a promising adjunctive strategy. In particular, stimulation of 5-HT_1_ receptors which negatively regulate serotonergic neuronal activity has been proposed as a strategy to manage l-DOPA-induced dyskinesia.

The 5-HT_1A_ and 5-HT_1B_ receptors belong to the GPCRs family and are coupled to Gi/o proteins. 5-HT_1A_ receptor is widely distributed throughout the CNS. It is expressed both presynaptically as an autoreceptor in the raphe nuclei and postsynaptically in cortical, hippocampal, and limbic regions of the brain responsible for modulation of mood, anxiety, and cognitive processes.^64^

On the other hand, 5-HT_1B_ receptors function mainly as presynaptic autoreceptors in the basal ganglia, striatum, and frontal cortex and may also act as a terminal heteroreceptor controlling the release of other neurotransmitters, such as acetylcholine, glutamate, dopamine, noradrenaline, and GABA.^64,65^

In PD models, aberrant activity of serotonergic neurons leads to the abnormal conversion of l-DOPA and the subsequent release of dopamine. Dual activation of presynaptic 5-HT_1A_ and 5-HT_1B_ autoreceptors dampens this activity, flattening dopamine spikes and reducing dyskinesia. Moreover, recent clinical findings indicate that Gα_0_-biased 5-HT_1A_ receptor agonist NLX-112 (clinical trial, phase III) was particularly effective in reducing levodopa-induced dyskinesia and motor symptoms in PD.^66,67^

Postsynaptic 5-HT_1A_ heteroreceptors in corticolimbic regions regulate neurotransmitters release by enhancing dopamine in the prefrontal cortex (PFC) and ventral tegmental area (VTA)^68,69^ while reducing striatal glutamate levels to facilitate motor control.^70^ Activation of 5-HT_1B_ heteroreceptors further disinhibits striatal dopamine release, promoting smoother motor output.^71^ Collectively, agonists targeting both 5-HT_1A_ and 5-HT_1B_ receptors offer a promising approach to rebalance motor loops and improve treatment tolerability.^66,72^

Eltoprazine, a dually acting agonist at 5-HT_1A_ and 5-HT_1B_ receptors, has been investigated for its potential to mitigate levodopa-induced dyskinesias (LIDs) in PD (Figure 7). LIDs are involuntary movements that can develop as a side effect of chronic levodopa therapy, which remains the primary treatment for PD.

**Figure 7:**
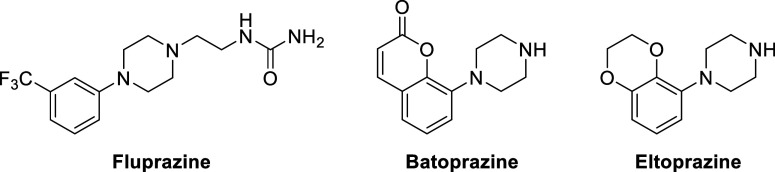
Structures of fluprazine, batoprazine, and eltoprazine—a dually acting serotonin 5-HT_1A_ and 5-HT_1B_ receptor agonist.

Among three arylpiperazine derivatives—eltoprazine, fluprazine, and batoprazine—eltoprazine has attracted the most attention. Initially identified as a nonsedative agent with antiaggressive properties, further development of these compounds was discontinued due to concerns regarding side effects and inconsistent efficacy.^73^

Eltoprazine was highly effective in reducing LID in animal models of PD: 6-hydroxydopamine (6-OHDA)-lesioned rats (0.3 mg/kg and 0.6 mg/kg) and 1-methyl-4-phenyl-1,2,3,6-tetrahydropyridine (MPTP)-treated macaques (0.6 mg/kg). However, this effect was accompanied by a partial attenuation of the therapeutic effect of l-DOPA.^74^ Interestingly, eltoprazine was found to synergistically potentiate the antidyskinetic effect of amantadine (eltoprazine 0.6 mg/kg plus amantadine 10 mg/kg).^74^ Further studies in MPTP-treated macaques revealed the administration of preladenant (5 mg/kg), a selective adenosine A_2A_ receptor antagonist, with eltoprazine (1 mg/kg) prevented the increased severity of parkinsonian motor symptoms but was unable to maintain a reduced expression of dyskinesia with repeated administration.^75^

A pivotal phase I/IIa randomized, double-blind, placebo-controlled, dose-finding study evaluated the safety and efficacy of single oral doses of eltoprazine (2.5 mg, 5 mg, and 7.5 mg) in 22 PD patients experiencing LIDs. Eltoprazine at 5 mg dose significantly reduced dyskinesia without compromising the beneficial motor effects of l-DOPA. Importantly, eltoprazine was well-tolerated, with the most common side effects being mild nausea and dizziness.^76^ A phase II clinical trial investigating eltoprazine in 60 patients with LID has been registered since 2016, but its current status is listed as “unknown”.^77^ Although it is uncertain whether this study will advance, the company Amarantus Bioscience Holdings, Inc. reported in 2019 that its subsidiary Elto Pharma Inc. had received a notice of allowance from the European Patent Office (EPO) for the use of eltoprazine—alone or in combination with other agents, including cannabidiol (CBD)—for the treatment of PD and LID.^78^

#### 3.2.2 Dually Acting 5-HT_1B_ Receptor Agonists/5-HT_6_ Receptor Antagonists: Preclinical Rationale toward Motor and Nonmotor Symptoms

Combining 5-HT_1B_ receptor activation with 5-HT_6_ receptor blockade represents yet another strategy to address both motor and nonmotor symptoms of PD. 5-HT_1B_ receptor agonists diminish l-DOPA-induced dyskinesia^79^ and exert antiparkinsonian-like effects in the rat models of PD, whereas antagonists of 5-HT_6_ receptor were reported to alleviate haloperidol-induced bradykinesia and catalepsy.^80,81^ Moreover, 5-HT_6_ receptor antagonists exhibit therapeutic potential in preclinical models of PD, likely owing to the high expression of 5-HT_6_ receptors in brain regions associated with motor control, such as the striatum.^6,82^ Thus, authors tested whether combination of 5-HT_1B_ receptor agonism with 5-HT_6_ receptor antagonism could preserve the therapeutic efficacy of l-DOPA while attenuate l-DOPA-induced motor fluctuations and simultaneously deliver procognitive and antidepressant-like effects.^83^

Using a pharmacophore-based design approach and merging the structural motifs responsible for intrinsic activity at 5-HT_1B_ receptor and antagonism at 5-HT_6_ receptor, a library of arylsulfonyl derivatives of isoindolines substituted with different alkyl amines was obtained.^84^ Synthesized compounds displayed high, nanomolar affinity for *h*5-HT_6_ receptor (*K*_i_ = 0.5–9.8 nM); moreover, selected representative derivatives exhibited potent antagonist properties at *h*5-HT_6_ receptor (*K*_b_ = 0.2–14 nM) (Figure 8).

**Figure 8:**
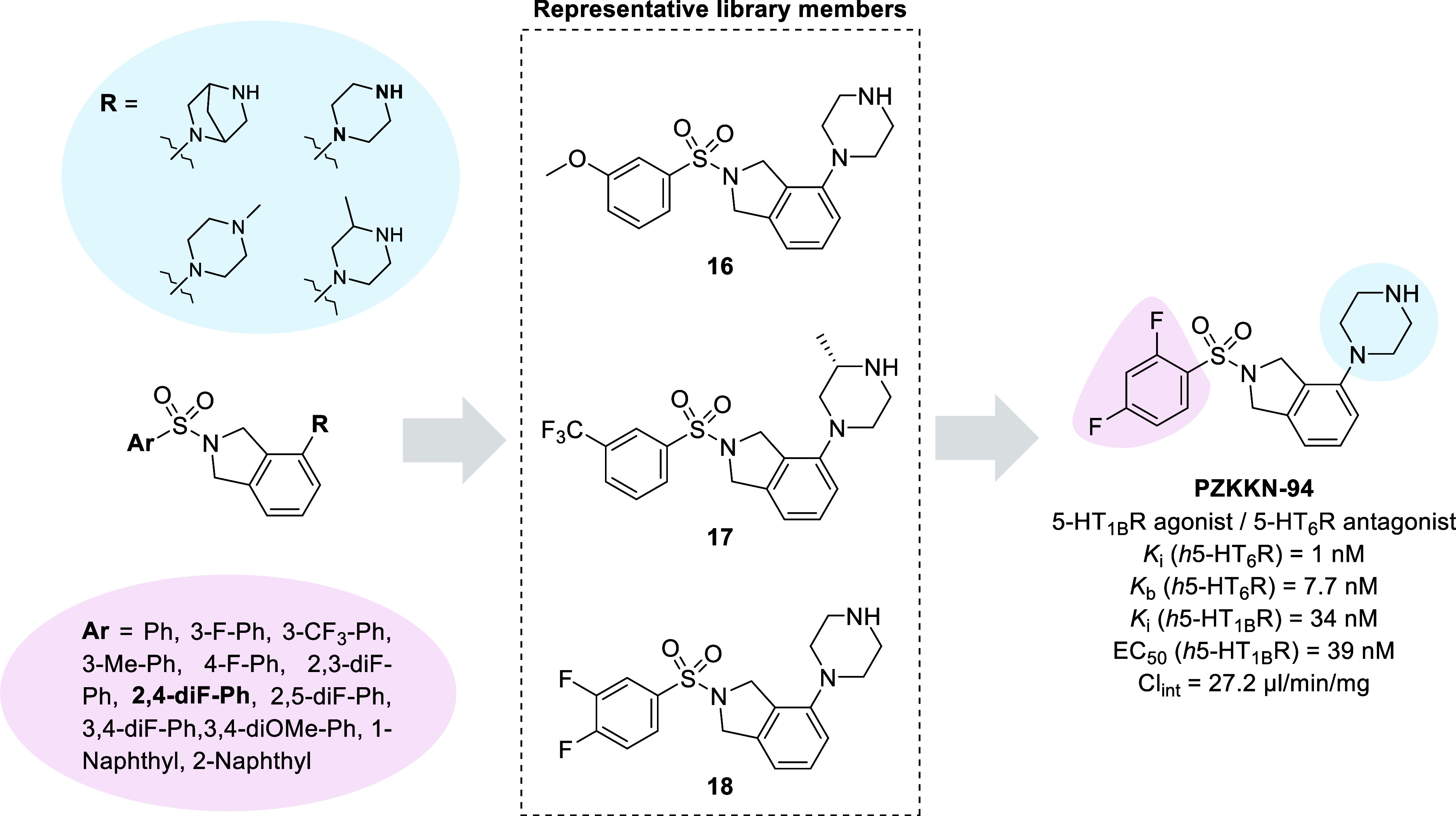
Example isoindoline derivatives **16**–**18** leading to the identification of dually acting 5-HT_1B_ receptor agonist/5-HT_6_ receptor antagonist PZKKN-94. *h*5-HT_1B_ receptor: [^3^H]-5-CT binding (CHO-K1 cells); *h*5-HT_6_ receptor: [^3^H]-LSD binding (HEK293 cells). Functional activity: G-protein dissociation Bioluminescence Resonance Energy Transfer (BRET) in HEK293T cells expressing respective receptor. Intrinsic clearance (Cl_int_) was measured in rat liver microsomes.^83,84^

Subsequent structural optimization aimed to improve metabolic stability, blood–brain barrier (BBB) permeability and safety led to the identification of compound PZKKN-94, difluoro substituted phenyl ring in combination with piperazine, as an amine fragment.^83^ PZKKN-94 was classified as a dually acting compound, functioning as a potent human 5-HT_1B_ receptor agonist (EC_50_ (*h*5-HT_1B_R) = 39 nM) and a selective 5-HT_6_ receptor antagonist (*K*_b_ = 7.7 nM). Importantly, PZKKN-94 exhibited approximately 10-fold greater agonist potency at rat and mouse 5-HT_1B_ receptor orthologs compared with the human 5-HT_1B_ receptor. This contrasts with the well-documented species-specific pharmacology of 5-HT_1B_ receptor, which is thought to arise from sequence variations that affect ligand binding and functional responses.^85^ Although such interspecies discrepancies often limit the translational value of preclinical models for 5-HT_1B_ receptor ligands, the fact that PZKKN-94 retained—and enhanced—potency in rodent receptors is advantageous. Unlike many 5-HT_1B_ ligands (e.g., sumatriptan and metergoline) that lose affinity in rodent models, PZKKN-94 ensures robust target engagement in preclinical species, facilitating reliable behavioral assessment. Furthermore, PZKKN-94 demonstrated nearly equipotent antagonist activity at rat and human 5-HT_6_ receptor orthologs, while showing approximately 10-fold lower potency at the mouse 5-HT_6_ ortholog.^83^

The antiparkinsonian potential of PZKKN-94 was demonstrated in two rat models: haloperidol-induced catalepsy and 6-OHDA-induced sensorimotor impairment. Importantly, it did not diminish the therapeutic effects of l-DOPA but significantly attenuated l-DOPA-induced motor complications, such as stepping and vibrissae test fluctuations, without influencing contralateral rotation, suggesting an absence of dopaminergic mimetic effects. Additionally, PZKKN-94 demonstrated procognitive effects, reversing learning deficits in the NOR task, enhancing cognitive flexibility in the attentional set-shifting assay, and producing antidepressant-like responses in the forced swim test. Although full therapeutic validation of this dual mechanism will require comprehensive elucidation of the intracellular signaling pathways engaged by 5-HT_1B_ and 5-HT_6_ receptors, the robust and convergent behavioral and pharmacological actions already demonstrated provide a compelling mechanistic basis for prioritizing this dual-acting strategy as a promising nondopaminergic therapeutic avenue in PD.

#### 3.2.3 Dually Acting 5-HT_2A_/5-HT_6_ Receptors Antagonists: Preclinical Evidence on Parkinson’s Disease-Related Dementia

Landipirdine (SYN-120) represents another example of drug targeting both 5-HT_2A_ and 5-HT_6_ receptors. The rational effort for its development stemmed from the procognitive potential associated with concurrent modulation of these receptors, prompting its evaluation for improving cognition in patients with PD.^86,87^ Initially developed by F. Hoffmann-La Roche Ltd., landipirdine’s evaluation was later pursued by Biotie Therapies Corp. and Acorda Therapeutics.^88^ Notably, beyond information disclosed in patent disclosure, detailed nonclinical pharmacological or functional characterization of landipirdine has been publicly released; nevertheless, the available documentation positions the compound as a dual 5-HT_2A_/5-HT_6_ receptor antagonist.^89^

Although no dedicated medicinal chemistry publications detail the SAR leading to the discovery of landipirdine, patent WO 2006/066790 A1 provides substantial insight into the structural modifications explored within tetraline derivatives (Figure 9).^90^

**Figure 9:**
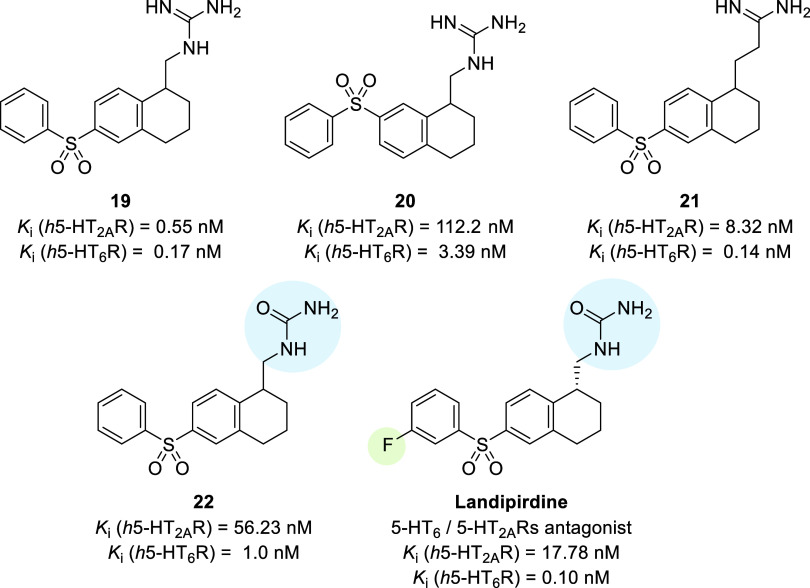
Examples of tetraline derivatives evaluated as potential dually acting 5-HT_2A_/5-HT_6_ receptors ligands. *h*5-HT_2A_ receptor: [^3^H]-ketanserin binding (CHO-K1 cells); *h*5-HT_6_ receptor: [^3^H]-LSD binding (HEK293 cells).^90^

The diversification strategy included the introduction of arylsulfonyl groups at positions 6 and 7 of the tetralin core, as well as the incorporation of substituted amine, urea, guanidine, or various heterocyclic moieties at position 1. Notably, the combination of a benzenesulfonyl group at position 6 with a guanidine moiety linked via a methylene bridge to the tetraline scaffold resulted in high affinity for both 5-HT_6_ and 5-HT_2A_ receptors (compound **19**). In contrast, shifting the benzenesulfonyl group to position 7 (compound **20**) reduced affinity for both targets. Substitution of the guanidine group with an amidine (compound **21**) maintained 5-HT_6_ receptor affinity but decreased 5-HT_2A_ receptor binding. Replacing the guanidine moiety with a urea group (compound **22**) diminished activity at both receptors. Interestingly, the introduction of a fluorine atom at the *meta* position of the benzenesulfonyl group enhanced 5-HT_2A_ receptor affinity relative to the unsubstituted analog and elevated 5-HT_6_ receptor affinity to subnanomolar levels. The (*R*)-enantiomer, later reported as landipirdine, was evaluated in clinical trials focusing on its potential to treat PD dementia.^86^

A notable phase II trial, the SYNAPSE trial (NCT02258152), on the safety, tolerability, and efficacy of landipirdine in patients revealed that while landipirdine was generally well-tolerated, it did not demonstrate significant improvements in cognitive assessments compared to the placebo. Although there were nominal improvements in cognitive activities of daily living and apathy scores,^91^ suggesting potential benefits that warrant further investigation, the development of landipirdine for cognition disorders and dementia was discontinued in 2020 and the compound became available for licensing.

## 4 Dual-Target Serotonergic Strategies in Psychotic Disorders

Psychotic disorders are a group of severe mental illnesses characterized by impaired reality testing. They typically manifest with delusions, hallucinations, disorganized thought and behavior, and perceptual disturbances. Another key domain includes negative symptoms such as social withdrawal, affective flattening, anhedonia, and reduced initiative and energy. Cognitive decline is also common, though not diagnostic. As a result, patients experience substantial functional impairment, social stigma, and reduced quality of life.^92^

Schizophrenia spectrum disorders have an idiopathic background resulting from a complex genetics–environment interplay, while other psychotic responses are usually secondary symptoms.

The integrated model explaining pathophysiology of idiopathic psychoses and schizophrenia combines several hypotheses and considers the interaction of glutamatergic, GABAergic, serotoninergic, and dopaminergic systems and regional differences in neurotransmitters. Briefly, it suggests that psychotic symptoms result from NMDA receptor hypofunction on cortical GABAergic interneurons, which, together with overactive 5-HT_2A_ receptors, leads to abnormal excitation of striatal dopaminergic neurons. Comorbid impairment in cortical neural activity further disturbs dopaminergic signaling in PFC, likely contributing to negative symptoms.^49,93^

### 4.1 Dually Acting 5-HT_2A_/5-HT_6_ Receptors Antagonists: Clinical Outcomes on Antipsychotic Effects and Cognition

Given this complex neurochemical background, targeting serotonergic receptors—particularly those involved in modulating glutamatergic and dopaminergic pathways—represents a promising strategy for developing novel antipsychotic agents. For instance, dually acting receptor antagonists such as idalopirdine, initially discovered by Eli Lilly as a selective 5-HT_6_ receptor antagonist and later developed by Lundbeck for AD (Figure 10), exemplify this approach. Similar to intepirdine, the rationale behind 5-HT_6_ receptor blockade was to enhance cholinergic and glutamatergic neurotransmission, potentially improving cognitive decline. Following early clinical signals suggesting procognitive effects, idalopirdine has been evaluated as an adjunctive therapy in schizophrenia, targeting cognitive and negative symptoms that are inadequately addressed by antipsychotics.

**Figure 10:**
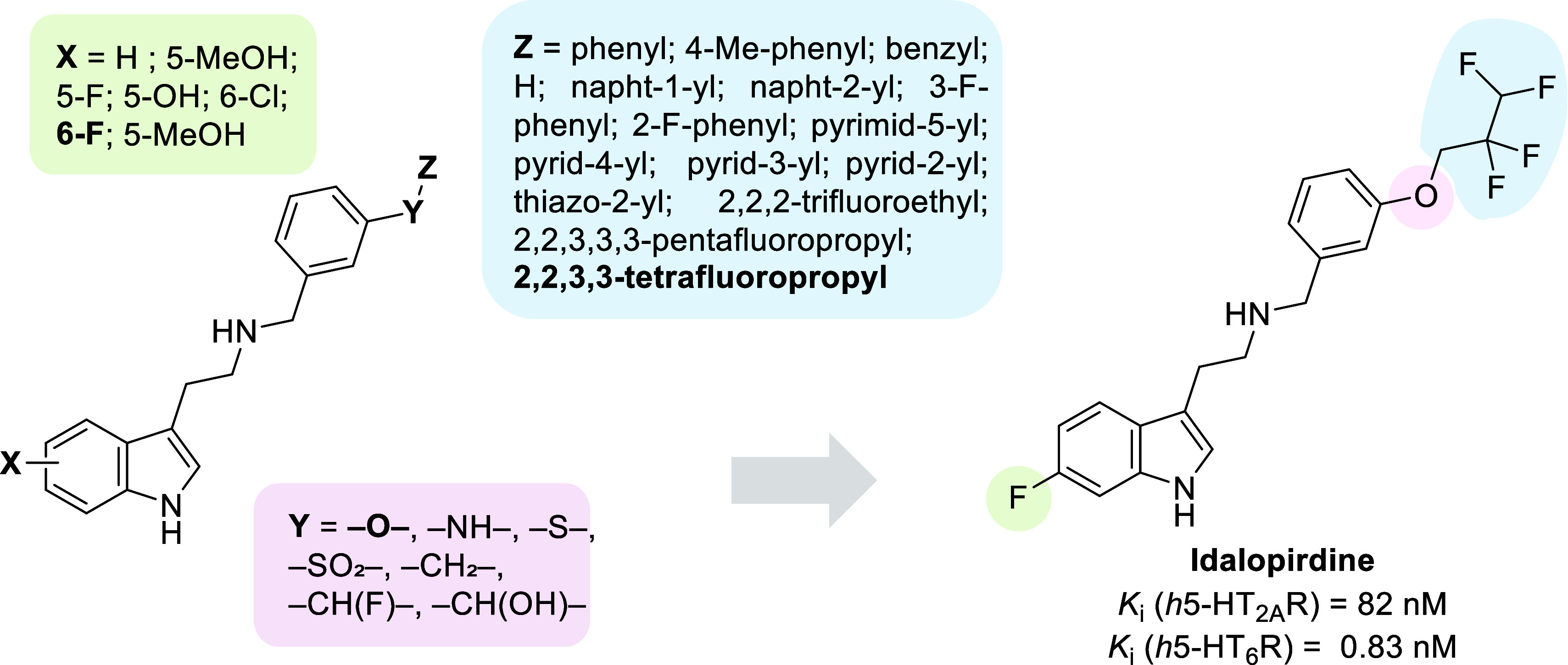
Patent of Eli Lilly discloses *N*-(2-arylethyl)­benzylamines as antagonists of the 5-HT_6_ receptor. *h*5-HT_6_ receptor: [^3^H]-LSD binding (BHK cells); *h*5-HT_2A_ receptor: [^125^I]-DOI binding (AV12 cells).^94^

The series of *N*-(2-arylethyl)­benzylamine derivatives disclosed in the Eli Lilly patent behave as antagonists of the 5-HT_6_ receptor.^94^ Although the patent does not present explicit affinity data, it highlights the indole moiety attached to the arylethyl fragment as the preferred core scaffold. Additionally, modifications involving halogen substitution on the indole ring—particularly fluorine at the 6 position—are identified as favorable and frequently observed among the synthesized compounds. Within the benzylamine fragment, *meta*-alkoxy substitution patterns on the benzyl ring emerge as common motifs, with short fluorinated alkoxy groups being among the most preferred substituents.

Idalopirdine is a 5-HT_6_ receptor antagonist (*K*_i_ = 0.83 nM, *K*_b_ = 4.9 nM), which additionally exhibits high affinity for adrenergic α_1A_ (*K*_i_ = 21 nM) and α_1B_ (*K*_i_ = 22 nM) receptors, and demonstrates moderate binding affinity for 5-HT_2A_ (*K*_i_ = 82 nM) receptor.^95^

The role of 5-HT_2A_ receptor antagonists and inverse agonists in the development of more efficient and safer antipsychotics has already been noted above (Section 2.1). The therapeutic potential of 5-HT_2A_ receptor, however, extends further. They are widely distributed in the brain—particularly in the cortex—where they modulate excitation–inhibition balance and regulate cortical feedback loops implicated in multiple CNS disorders.

Animal studies showed that 5-HT_2A_ receptor knockout mice display reduced depressive- and anxiety-like behaviors, consistent with antidepressant- and anxiolytic-like effects of their antagonists.^96^ Previous clinical evidence suggested that nonselective 5-HT_2A_ antagonists enhanced SSRI efficacy in depression and obsessive-compulsive disorder,^97^ while the recent CLARITY trial (NCT03018340) demonstrated that the pimavanserin potentiated SSRI and serotonin-norepinephrine reuptake inhibitors (SNRI) efficacy in major depression.^98^

In a rat model, idalopirdine administered as monotherapy mitigated cognitive deficits induced by subchronic PCP exposure, supporting its possible application in the management of schizophrenia-related cognitive impairment.^95^ Fijał et al.^99^ demonstrated that clozapine, but neither the selective 5-HT_6_ receptor antagonists SB-271046 or SB-399885 nor the 5-HT_2A_ receptor antagonist M100907 alone, reversed dizocilpine-induced prepulse inhibition (PPI) deficits in rats. However, combined administration of SB-271046 or SB-399885 with M100907 effectively restored PPI. Notably, all these compounds potentiated the effects of subeffective doses of clozapine.^99^

Idalopirdine was investigated in a clinical trial as a monotherapy for schizophrenia, involving a small group of 20 patients who received escalating doses (60/180 mg or 120/240 mg) or placebo once daily over a 14 day period. Cognitive function was evaluated using the Brief Assessment of Cognition in Schizophrenia (BACS), where a dose-related improvement was observed, reaching statistical significance at the highest dose of 240 mg.^100^ No cognitive enhancement was noted in the placebo group.

In a separate clinical trial (NCT00810667), idalopirdine was tested as an add-on therapy to risperidone, administered at 60 mg twice daily for 12 weeks. Inconsistent with the preclinical data (see ref 95), in this setting, idalopirdine did not show any significant benefit over placebo in reducing schizophrenia symptoms, as measured by the Positive and Negative Syndrome Scale (PANSS), nor did it produce notable changes in BACS or the PANSS cognitive subscale.^101^ Despite these findings, idalopirdine was generally well-tolerated, though no further studies in schizophrenia patients have been reported.

More recently, attention has shifted to psychedelic-induced neuroplasticity mediated by activated 5-HT_2A_ receptor.^102,103^ Current studies indicate that selective modulation of specific 5-HT_2A_ signaling pathways may decouple the psychedelic, antidepressant, and antipsychotic effects of 5-HT_2A_ ligands, particularly those that are functionally selective and target distinct receptor-operated signaling cascades. This emerging understanding offers new insights into the role of 5-HT_2A_ receptors in the pathophysiology and potential treatment of psychiatric disorders.^104,105^

### 4.2 Dually Acting 5-HT_3_/5-HT_6_ Receptors Antagonists: Preclinical Evidence for Antipsychotic and Procognitive Effects

The complex neurochemical disturbances underlying schizophrenia have prompted the exploration of multitarget pharmacological strategies. Among these, dually acting 5-HT_3_/5-HT_6_ receptors antagonists have emerged as promising candidates, given their potential to address both the serotonergic dysregulation and cognitive deficits characteristic of the disorder.

The 5-HT_3_ receptor is an ionotropic receptor which belongs to the pentameric ligand-gated ion channel superfamily. The 5-HT_3_ receptor is distributed both in the peripheral and CNS, in brain regions like the hippocampus, putamen, caudate nucleus, and amygdala and in the peripheral nervous system in the gastrointestinal tract. On presynaptic nerve terminals, 5-HT_3_ receptors regulate calcium influx, which in turn modulates the release of neurotransmitters like dopamine, GABA, and acetylcholine. On postsynaptic GABAergic interneurons, the receptor is involved in fast excitatory depolarization by allowing the influx of sodium and potassium ions. Blocking presynaptic 5-HT_3_ receptors inhibits excessive mesolimbic dopamine activity and GABA release and enhances acetylcholine signaling in the hippocampus and cortex.

Ondansetron and granisetron, antagonists of the 5-HT_3_ receptor, primarily used as antiemetics, have shown beneficial effects as adjunctive therapies in schizophrenia, notably by improving negative symptoms and cognitive decline. Additionally, 5-HT_3_ receptor antagonists reduced extrapyramidal side effects (such as catalepsy and tardive dyskinesia) induced by haloperidol and 5-hydroxytryptophan. Notably, an analysis of the pharmacological profile of clozapine, the only antipsychotic used for treatment-resistant schizophrenia, revealed that, in addition to blocking 5-HT_2A_ receptor, clozapine also antagonizes 5-HT_3_ and 5-HT_6_ receptors. This combination of receptor modulatory effects alongside the established cognitive-enhancing properties of 5-HT_6_R antagonists inspired Zajdel, Lamaty, Popik, and collaborators to develop dually acting 5-HT_3_/5-HT_6_ receptor antagonists as a potential treatment for schizophrenia. Employing a hybrid approach, they developed new arylsulfonamide derivatives of 1*H*-pyrrolo­[3,2-*c*]­quinoline derivatives.^106^

The SAR analysis of obtained derivatives revealed that introduction of piperazine or methyl piperazine at C4 of the heterocyclic scaffold as well as monosubstitution at the *meta* position of the arylsulfonyl fragment affords optimal antagonist properties at both targets (Figure 11). Moreover, a fused benzene ring in the tricyclic core is compulsory for the antagonist activity at 5-HT_3_ receptor.

**Figure 11:**
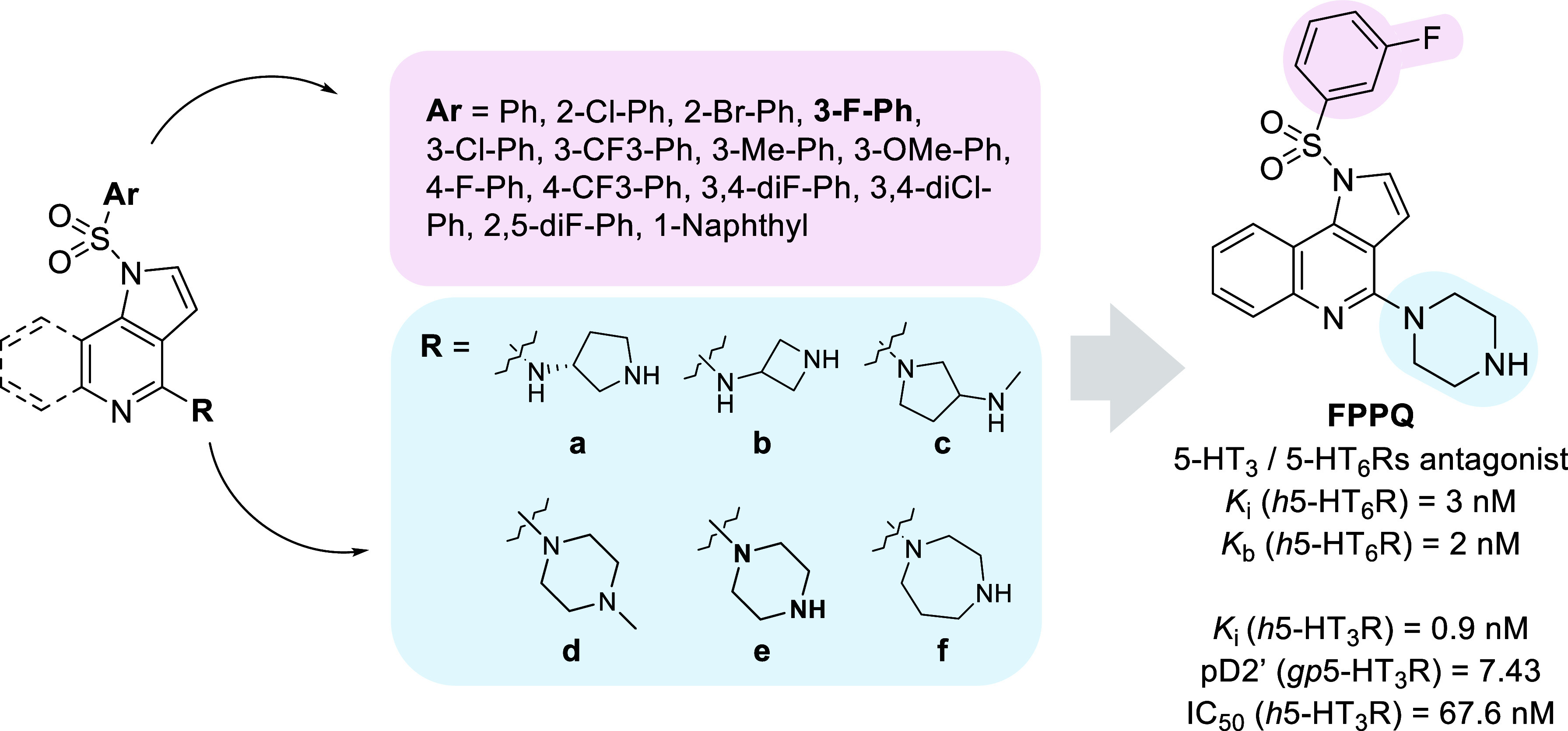
SAR analysis in a group of arylsulfonamide derivatives of 1*H*-pyrrolo­[3,2-*c*]­quinoline leading to the identification of dually acting 5-HT_3_ receptor/5-HT_6_ receptor antagonist FPPQ. 5-HT_6_ receptor: [^3^H]-LSD binding (HEK293 cells) and cAMP functional antagonism (1321N1 cells). 5-HT_3_ receptor: [^3^H]-BRL 43694 binding (HEK293 cells); antagonism: *ex vivo* guinea pig ileum and IonFlux electrophysiology (CHO-K1 cells).^107^

The studies led to the identification of compound FPPQ—the first-in-class dually acting ligand, functioning as both a 5-HT_3_ receptor antagonist and a neutral antagonist of the 5-HT_6_ receptor in the Gs pathway. FPPQ displayed balanced low nanomolar affinity at both receptors, selectivity over 87 targets, decent brain penetration, and a safety profile with no propensity to evoke off-target-related side effects. The *in vitro* activity of FPPQ translated well into results from animal studies since FPPQ displayed an antipsychotic-like effect by inhibiting PCP-induced hyperactivity and procognitive properties in the NOR test (MED = 1 mg/kg) in male Sprague–Dawley rats.

Unlike FPPQ, neither SB-399885 (a 5-HT_6_ receptor inverse agonist) nor CPPQ (a 5-HT_6_ receptor neutral antagonist) as well as ondansetron (a 5-HT_3_ receptor antagonist) reversed PCP-induced hyperactivity. Notably, coadministration of ondansetron (a 5-HT_3_ receptor antagonist) with CPPQ—but not with SB-399885—significantly attenuated PCP-induced hyperactivity in rats. These findings suggest that the combination of 5-HT_3_ receptor antagonism and 5-HT_6_ receptor neutral antagonism contributes to alleviating the positive-like symptoms of psychosis.

### 4.3 Dually Acting 5-HT_2A_/5-HT_2C_ Receptors Inverse Agonists: Preclinical Evidence of Antipsychotic Effects

Dual modulation of 5-HT_2A_ and 5-HT_2C_ receptors presents a promising approach for the treatment of dementia-related psychosis (DRP), particularly in minimizing the side effects associated with traditional dopamine D_2_ receptor antagonists. While pimavanserin which behaves as an inverse agonist at the 5-HT_2A_ receptor-operated Gα_i_1 signaling pathway/neutral antagonist at the 5-HT_2A_ receptor-operated canonical Gq/11 pathway and inverse agonist at 5-HT_2C_ receptor had demonstrated clinical utility, its limitations in efficacy and liability for *h*ERG-mediated cardiotoxicity motivated further medicinal chemistry efforts.^18,108^ Oguma et al. approached this challenge by systematically modifying the pimavanserin scaffold to develop a dual 5-HT_2A_/5-HT_2C_ receptors inverse agonist with an optimized safety and pharmacological profile (Figure 12).^109^

**Figure 12:**
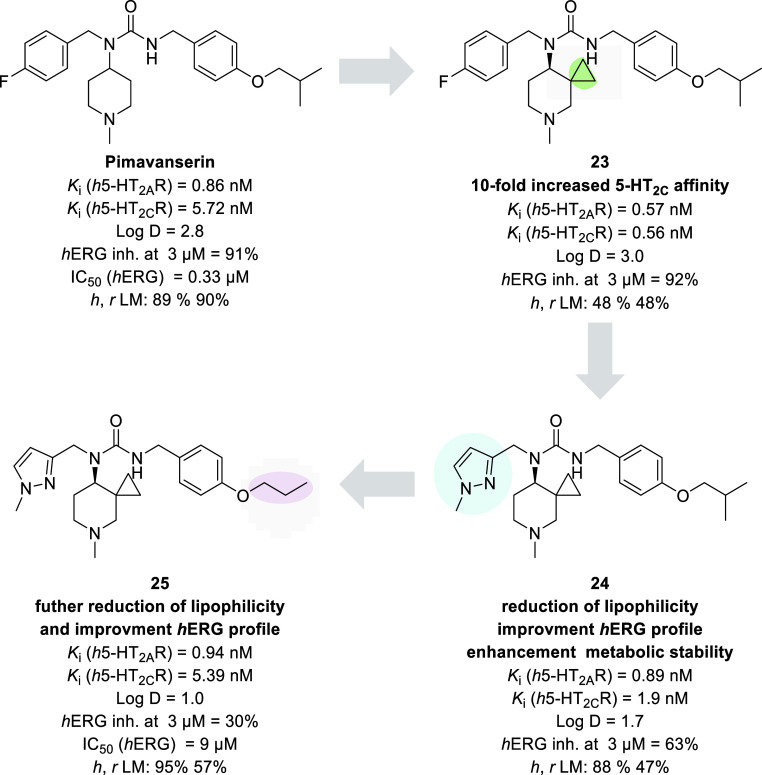
Stepwise structural optimization of pimavanserin leading to the discovery of compound **25**. *h*5-HT_2A_ receptor: [^3^H]-ketanserin binding (HEK293 cells); *h*5-HT_2C_ receptor: [^3^H]-mesulergine binding (HEK293 cells); Log *D* determined in 1-octanol/phosphate buffer at pH 7.4; *h*ERG inhibition at 3 μM and IC_50_ (*h*ERG): automated whole-cell patch clamp (CHO cells); *h*, *r* LM: % remaining after 30 min incubation with human and rat liver microsomes.^109^

The initial structural modification involved the incorporation of a spirocyclopropyl group into the piperidine moiety (compound **23**), which significantly enhanced 5-HT_2C_ receptor affinity—resulting in a 10-fold increase—without compromising 5-HT_2A_ receptor potency.

Further optimizations included replacing the *para*-fluorophenyl moiety with a heteroaromatic group (compound **24**) to reduce lipophilicity and enhance metabolic stability. An additional key modification entailed replacing the isobutyl group with *n*-propyl, thereby improving the compound’s physicochemical profile. These concerted changes culminated in the discovery of compound **25**, which demonstrated balanced nanomolar affinity at both 5-HT_2A_ and 5-HT_2C_ receptors, no affinity for human D_2_ receptor (*K*_i_ > 2 μM) as well as for other monoamine GPCRs such as 5-HT_1A_ receptor, histaminergic H_1_ receptor, and adrenergic α_1A_, α_2A_, α_2B_, or α_2C_, minimal *h*ERG inhibition, and improved pharmacokinetic properties. Of note, cocrystal structures provided key ligand–receptor interactions: pimavanserin was resolved in complex with 5-HT_2A_ receptor, while the spirocyclopropyl analogue (compound **23**) was resolved with 5-HT_2C_ receptor. Collectively, these structural insights rationalized the design strategy and highlighted the critical binding determinants supporting the observed potency and selectivity within this scaffold series. Functionally, compound **25** produced antipsychotic-like effects in rodent models of hyperactivity without inducing extrapyramidal symptoms, reinforcing the therapeutic value of dual inverse agonism at these serotonin receptors as a mechanistically differentiated strategy for treating DRP. *In vivo*, compound **25** (3 and 10 mg/kg, *sc*) significantly suppressed MK-801-induced hyperactivity in male Wistar rats, with robust efficacy already at 3 mg/kg, while also engaging cortical 5-HT_2A_ and modulating dopamine release as a functional marker of 5-HT_2C_ involvement.

### 4.4 Dually Acting TAAR1 Agonists/5-HT_1A_ Receptors Partial Agonists: Clinical Evidence on Antipsychotic Efficacy

Recent research emphasizes the potential of targeting alternative, nondopaminergic mechanisms to develop more effective antipsychotic therapies with fewer side effects. Among these, the trace amine-associated receptor 1 (TAAR1) has emerged as a promising candidate. TAAR1 is an intracellular GPCR activated by endogenous trace amines such as β-phenylethylamine, tyramine, or tryptamine, which exert feedback control over monoaminergic neurotransmission.^110^ Although data on its distribution are limited, TAAR1 is located in brain regions implicated in schizophrenia, including the VTA, DRN, and PFC. Preclinical studies have showed that TAAR1 agonists suppress midbrain dopaminergic and serotonergic activity while enhancing prefrontal glutamatergic function, thereby producing antipsychotic-like effects in animal models.^111^ These findings are supported by clinical data linking the TAAR1 partial loss-of-function variant with schizophrenia, further underscoring its therapeutic relevance.^112^

An example of neuropsychiatric drug development involving nondopaminergic pathways to achieve antipsychotic efficacy is exemplified by ulotaront (SEP-363856, also known as SEP-856, Figure 13).^114^ This first-in-class compound acts as an agonist of TAAR1 (EC_50_ = 38 nM)^113^ and 5-HT_1A_ (*K*_i_ = 0.284 μM, EC_50_ = 2.3 μM) receptor, without significant affinity for D_2_ receptor (*K*_i_ = 21.3 μM).^115^ The 5-HT_1A_ receptor agonism is thought to modulate serotonergic and dopaminergic neurotransmission, potentially enhancing dopamine release in the prefrontal cortex and improving symptoms of schizophrenia and related disorders. Such a receptor profile partially explains the observed clinical efficacy of ulotaront and highlights the promising therapeutic potential of 5-HT and TAAR1 modulation in the development of next-generation antipsychotics. The SAR study around ulotaront and related analogs highlights a highly constrained pharmacophore, where small structural changes significantly alter TAAR1 agonist potency.^113^

**Figure 13:**
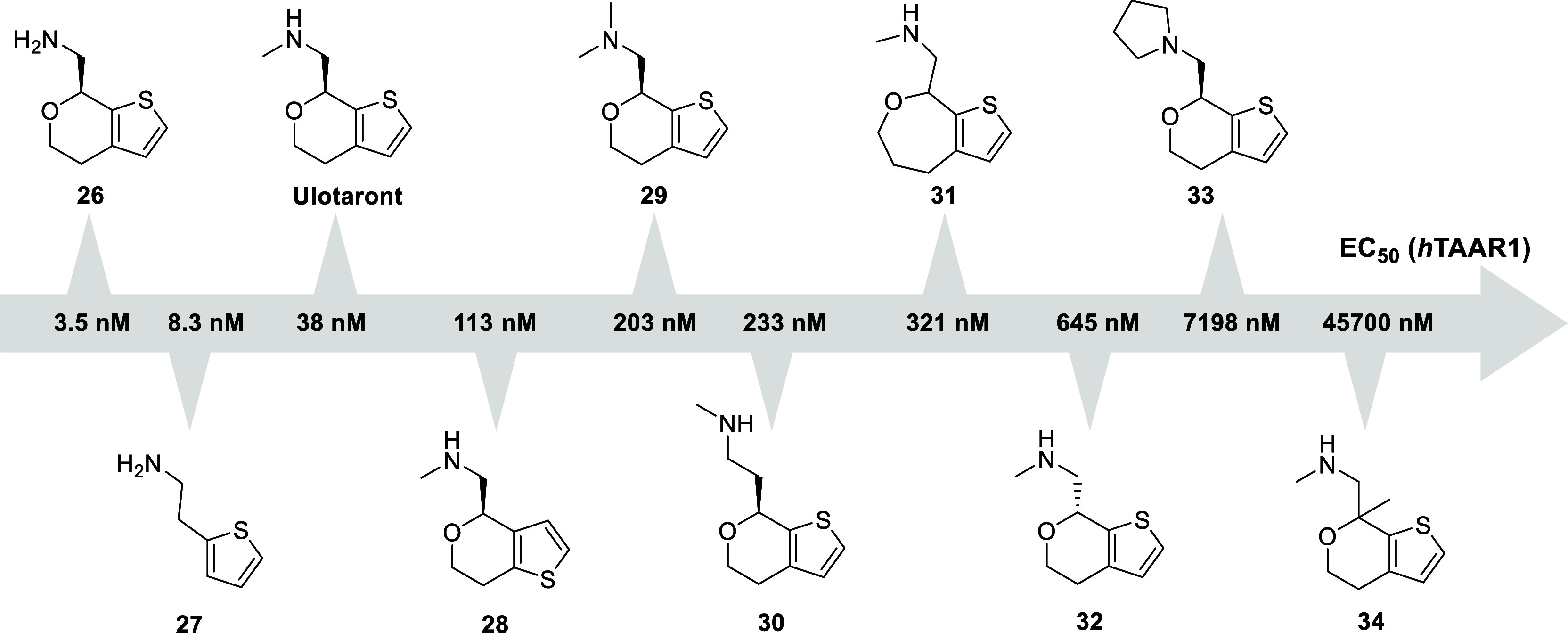
Human TAAR1 agonistic activity of ulotaront and its analogs. *h*TAAR1: cAMP functional agonism (CHO-K1 cells).^113^

Ulotaront, the (*S*)-enantiomer, is approximately 17-fold more potent than the (*R*)-enantiomer (compound **32**), reflecting a stereoselective interaction with the TAAR1 binding site. Removal of the *N*-methyl group (compound **26**) increased potency by approximately 11-fold, possibly by enabling an additional polar interaction within the binding site, while *N,N*-dimethylation (compound **29**) reduced activity, suggesting steric or conformational interference. Substitution at the chiral center, such as adding a methyl group (compound **34**), dramatically decreased TAAR1 potency by over 1000-fold. Modifications to the amine moiety also affected activity; extension of the linker from one to two carbons (compound **30**) caused a 6-fold drop in potency, and incorporation into a pyrrolidine ring (compound **33**) further reduced efficacy, suggesting a tight spatial requirement between the amine and the receptor. The size and conformation of the fused oxygen-containing ring system are equally critical; expanding the dihydropyran ring to a tetrahydrooxepin ring (compound **31**) leads to a 6-fold potency decrease when compared to ulotaront. The position of the sulfur atom in the fused thiophene ring influenced receptor binding; shifting it to the 2 or 3 position (compound **28**) reduced potency by approximately 6-fold and 3-fold, respectively. Interestingly, ring-opened analogs such as thiophen-2-ylethanamine (compound **27**) and thiophen-3-ylethanamine retained high activity, with the former—structurally corresponding to ulotaront—being about three times more potent than its isomer. Collectively, these findings demonstrate that TAAR1 agonism of ulotaront depends on precise stereochemistry, amine configuration, linker length, and fused ring topology.

Clinically, ulotaront has demonstrated antipsychotic efficacy without direct blockade of D_2_ receptor. In a randomized phase II trial, it showed significant symptom improvement compared to placebo, with sustained benefit and favorable tolerability in the open-label extension.^114,116^ However, two phase III trials in acute schizophrenia did not meet the primary endpoints. Despite this, the program retains FDA Breakthrough Therapy designation, and further phase III trials in schizophrenia and a phase II/III program in major depressive disorder are currently ongoing.^117^

### 4.5 Dually Acting TAAR1/5-HT_2C_ Receptors Agonists: Preclinical Evidence on Psychosis Symptoms (Positive, Negative, and Cognitive)

Building on promising phase II and phase III clinical trial results of the TAAR1 agonist/5-HT_1A_ receptor partial agonist ulotaront in schizophrenia, Lu et al. conducted detailed SAR studies aimed at developing dually acting TAAR1/5-HT_2C_ agonists, potentially addressing a broader range of symptoms associated with schizophrenia and related disorders.^118^ The initial series involved minor modifications to ulotaront, including substitutions on the dihydrofuran moiety and alterations to the amine methyl group. However, these changes did not significantly enhance potency. The second series involved breaking the dihydropyran ring of ulotaront, leading to compound **37** (Figure 14), which maintained comparable TAAR1 agonism to ulotaront, but exhibited enhanced activity at 5-HT_2C_ receptor. The most successful series involved aza-tricyclization, resulting in compound **36**. This compound demonstrated superior potency, with an EC_50_ of 0.022 μM and *E*_max_ of 95.255% for TAAR1 agonism, alongside a remarkable >1200-fold increase in 5-HT_2C_ receptor agonism compared to ulotaront. The SAR studies revealed that the tricyclic ring system is optimal for TAAR1 agonism. Moreover, the position of the thienyl sulfur is crucial in maintaining TAAR1 agonistic activity, as it enables an additional π–sulfur interaction with aromatic features of the binding site. Comprehensive *in vivo* studies confirmed its favorable pharmacological profile: following oral administration, compound **36** exhibited advantageous pharmacokinetics, rapid brain penetration, and sustained exposure in the central nervous system. Behavioral testing demonstrated efficacy in attenuating MK-801- (0.8 mg/kg, *ip*) and methamphetamine-induced (3 mg/kg, *ip*) hyperlocomotion in male C57BL/6J mice at doses 3 and 10 mg/kg (*po*). It further inhibited DOI-evoked head-twitch responses (5 mg/kg, *ip*) at oral doses 1 and 10 mg/kg. In the chronic social defeat stress (CSDS) model in C57BL/6J mice, **36** reversed social avoidance behavior (ED_50_ of 2.81 mg/kg, *po*), indicating efficacy against negative-like symptoms. Cognitive benefits were demonstrated in the NOR test, where compound **36** (1 mg/kg, *po*) improved memory performance in both healthy C57BL/6J mice and APP/PS1 transgenic mice modeling AD. In addition, pharmacokinetic studies in Sprague–Dawley rats confirmed efficient brain penetration and high brain-to-plasma ratios, while repeated-dose toxicology assessments in rats (2.5 or 7.5 mg/kg/day) and beagle dogs (0.75 or 2.5 mg/kg/day) showed good tolerability. Cardiovascular safety evaluations in cynomolgus monkeys demonstrated no prolongation of QT/QTc intervals, although a mild reduction in body temperature was observed. Collectively, the dual TAAR1/5-HT_2C_ agonism exhibited by **36** translates into antipsychotic-, antidepressant-, and procognitive-like effects *in vivo*, highlighting this compound as a next-generation candidate for the treatment of schizophrenia and related disorders.

**Figure 14:**
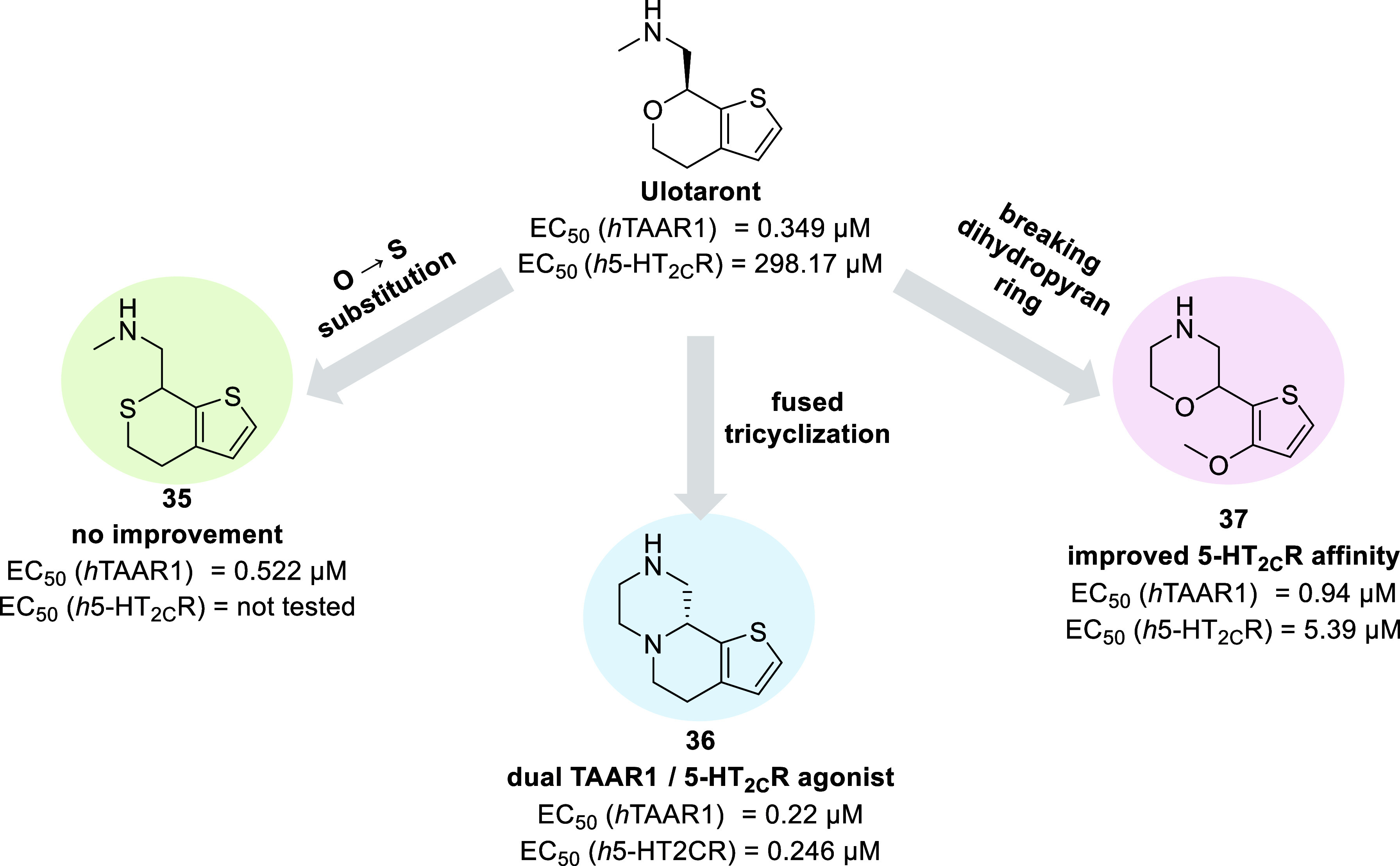
Representative potent compounds derived from ulotaront through distinct optimization strategies. *h*TAAR1: cAMP functional agonism (CHO-K1 cells); *h*5-HT_2C_ receptor: functional activity FLIPR calcium assay (HEK293 cells).^118^

## 5 Dual Serotonergic Approaches in Mood Disorders

Depression and anxiety frequently co-occur, yet background pathophysiology is not fully understood. Evidence points to monoaminergic dysfunction, particularly impaired 5-HT neurotransmission, culminating in maladaptive neuroplasticity. In a simplified model, excessive 5-HT_1A_ presynaptic autoreceptor activity in the DRN reduces serotonergic firing, while 5-HT_1B_ terminal autoreceptors further curb 5-HT release in target regions, together diminishing activation of cortical and hippocampal postsynaptic (hetero) 5-HT_1A_ receptors.^119^ This view has been supported by a number of pharmacological and genetic animal models,^96^ as well as clinical finding.^120,121^ Going further, current preclinical research indicates that biased 5-HT_1A_ agonists favoring activation of postsynaptic heteroreceptors produced stronger antidepressant-like effects, whereas agonists targeting 5-HT_1A_ somatodendritic autoreceptors and 5-HT_1B_ antagonists reduce anxiety.^122,123^

Prior research demonstrated that 5-HT_2A_ receptors blockade increases cortical excitatory feedback and enhances 5-HT release, contributing to antidepressant and anxiolytic efficacy. Similar effects were reported for 5-HT_2C_ receptor antagonists, which disinhibits mesocorticolimbic dopamine and noradrenaline signaling.^48,49,96^ As previously noted, agonism at 5-HT_2A_ receptors confers antidepressant efficacy of psychedelics, proposed to be driven via NMDA- and glutamate-dependent cortical and hippocampal synaptic plasticity.^104,105^ Although contrasting, these findings may converge on paradoxical long-term 5-HT_2A_ and 5-HT_2C_ receptors downregulation/internalization, induced by both receptor’s agonists and antagonists.^124^

Research demonstrated antidepressant and procognitive effects of 5-HT_3_, 5-HT_6_, and 5-HT_7_ receptors antagonists, potentially conveyed by enhanced cortical and hippocampal ACh release and balanced mesocorticolimbic dopamine signaling.^8^ Conversely, human positron emission tomography (PET) imaging studies linked low 5-HT_4_ receptors binding with anxiety and depression.^125^

Collectively, distinct 5-HT receptor subtypes differentially contribute to depression and anxiety, supporting the development of dually acting ligands as a promising therapeutic strategy.

### 5.1 Dually Acting 5-HT_1A_/5-HT_2C_ Receptors Agonists: Antidepressant and Anxiolytic Mechanisms

As noted above, antidepressant and anxiolytic efficacies are typically related to 5-HT_2A_ and 5-HT_2C_ antagonism. However, there are also contrasting studies, linking 5-HT_2C_ agonism with similar effects.^126^

Based on current knowledge, activation of 5-HT_2C_ receptors generally does not affect 5-HT release yet can produce an anxiogenic effect dependent on the recruitment of the stress axis and corticotropin-releasing hormone (CRH) release, which in turn increases 5-HT neurons firing in the DRN. Only then, an excessive 5-HT level activates 5-HT_2C_ receptors on GABAergic interneurons, providing negative feedback on 5-HT release. Thus, 5-HT_2C_ agonists, by increasing stress-resilience, may produce similar effects to their antagonists. However, the reduced 5-HT signaling again impairs postsynaptic 5-HT_1A_ heteroreceptors activation, so their external agonism can sustain the antidepressant component.^126^

Another possible mechanism depends on the previously mentioned 5-HT_2C_ receptors downregulation.^124^ Notably, agonists of 5-HT_2C_ receptor produce an anorectic effect, as demonstrated clinically with lorcaserin,^127^ which may benefit selected patient populations.

Against this mechanistic background, arylmorpholines constitute a privileged CNS scaffold not only for norepinephrine transporter (NET) inhibitors (e.g., reboxetine) but also due to the favorable interaction with serotonergic receptor, particularly 5-HT_2C_ and 5-HT_1A_. Derivatives of 2-(3-trifluoromethyl)­phenyl morpholine were disclosed in U.S. Patent 3637680 by Mauvernay et al. in 1972 as compounds with tranquilizing effect on the CNS as well as anti-inflammatory and analgesic activity.^128^ Although detailed data on the SAR in this group of compounds is not provided in the literature, the patent provides insight into applied structural modifications, which involved introduction of various aryl, alkyl, and alicyclic fragments on the nitrogen atom of the morpholine moiety (Figure 15).

**Figure 15:**
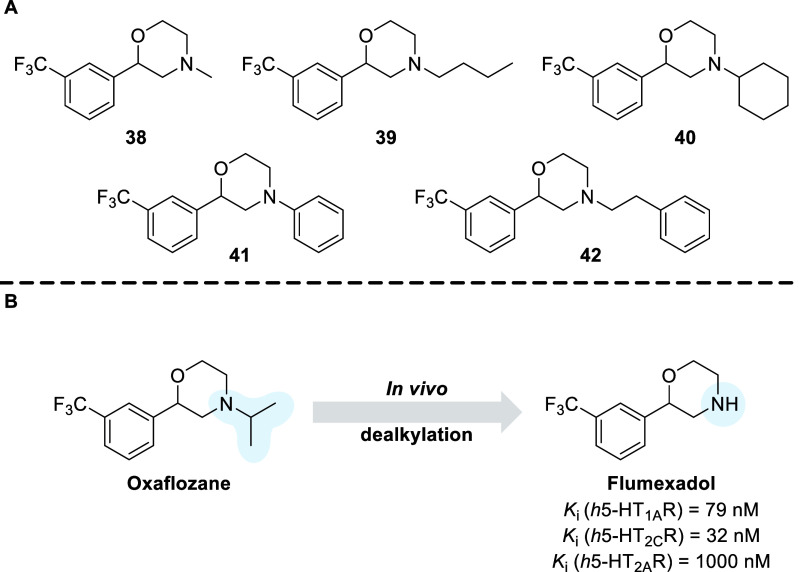
(A) Structural modifications in a group of 2-(3-trifluoromethyl)­phenyl morpholine derivatives leading to the identification of oxaflozane. (B) Hepatic metabolism of oxaflozane leading to flumexadol.^128^

Later pharmacological studies, including those by Busch and colleagues in 1976, identified the 2-(3-trifluoromethyl)­phenyl morpholine derivative bearing the isopropyl substituent (oxaflozane) and laid the groundwork for its clinical development.^129^ Marketed in France under the trade name *Conflictan* in the early 1980s, oxaflozane was primarily used as an anxiolytic and antidepressant; however, its use was later discontinued.

Oxaflozane acts as a prodrug, undergoing hepatic dealkylation to yield flumexadol, an active metabolite with agonist properties as 5-HT_1A_ and 5-HT_2C_ receptors. This receptor profile suggests a mechanism distinct from traditional tricyclic antidepressants, aligning it more closely with serotonergic agents. Of note, the (+)-enantiomer of flumexadol demonstrated high affinity for the 5-HT_2C_ receptor (*K*_i_ = 25 nM) and exhibited approximately 40-fold selectivity over the 5-HT_2A_ receptor in binding assays.^130^ The racemic form was initially described for its analgesic properties, with no mention of activity at the 5-HT_2C_ receptor.^131^ These findings suggest that flumexadol may have potential utility not only as an analgesic but also as an anorectic agent.^130^

### 5.2 Dually Acting 5-HT_2A_/5-HT_7_ Receptors Antagonists: Mood Regulation and Circadian Effects

Emerging evidence supports a dual-antagonism strategy targeting the 5-HT_2A_ and 5-HT_7_ receptors to treat mood disorders. Antagonism at 5-HT_2A_ receptor contributes to the therapeutic profile of several antipsychotics and antidepressants, while selective 5-HT_7_ receptor blockade produces antidepressant-like effects in preclinical models and may produce procognitive effects in animal models.^132–134^ The 5-HT_7_ receptor, the most recently identified serotonin subtype, belongs to the GPCR family and is positively coupled to Gs protein, increasing the level of cAMP upon activation. Of note, 5-HT_7_ receptor exhibits a high level of constitutive activity at the Gs signaling pathway and is expressed in the cortex, thalamus, hypothalamus, and striatum and is implicated in circadian rhythms regulation. 5-HT_7_ receptor is considered as a treatment approach for depression^135^ and mood and anxiety disorders.^136^ Pharmacological blockade with agents such as JNJ-18038683 attenuates both photic and nonphotic phase shifts in rodents, consistent with a role in the suprachiasmatic clock.^137^ The same selective 5-HT_7_ receptor antagonist has progressed to phase II clinical trials for depression treatment.^138^ Clinically, drugs with meaningful 5-HT_2A_/5-HT_7_ receptors activity illustrate the translational potential of this approach: lurasidone exhibits high affinity antagonism at 5-HT_7_ receptor alongside 5-HT_2A_ receptor blockade and shows efficacy in bipolar depression, while vortioxetine combines 5-HT_7_ receptor antagonism with multimodal serotonergic actions and is effective in major depressive disorder.^14^ Together, these data justify continued development of compounds that jointly target 5-HT_2A_ and 5-HT_7_ receptors, with opportunities to extend investigation toward circadian/sleep endpoints, cognition, and biomarkers of target engagement.

To achieve this dual antagonism, Dvorak and colleagues at Janssen Research & Development, LLC, synthesized and identified a series of 2-alkyl-3-phenyl-2,4,5,6,7,8-hexahydropyrazolo­[3,4-*d*]­azepines (Figure 16).

**Figure 16:**
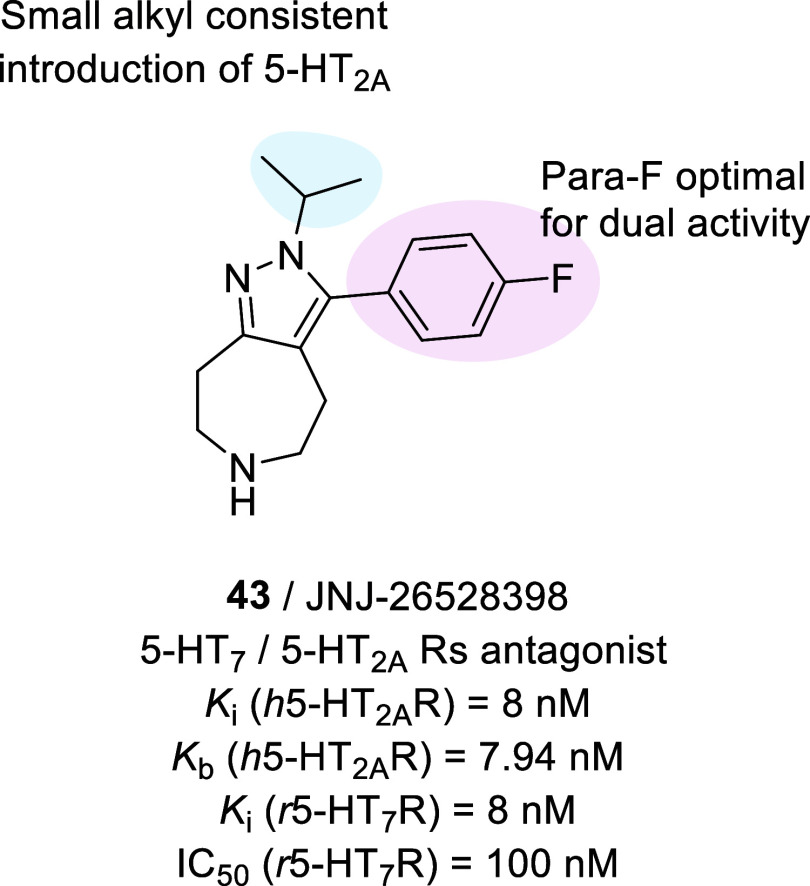
Key structure–activity relationship insights for 2-alkyl-3-phenyl-2,4,5,6,7,8-hexahydropyrazolo­[3,4-*d*]­azepine derivatives. *h*5-HT_2A_ receptor: [^3^H]-ketanserin binding (CHO cells) and calcium mobilization functional antagonism (CHO-K1 cells); *r*5-HT_7_ receptor: [^3^H]-5-CT binding (HEK293 cells) and cAMP functional assay (HEK293 cells).^139^

These compounds were designed to have high affinity for both 5-HT_2A_ and 5-HT_7_ receptors.^139^ The SAR studies focused on optimizing compounds for dual 5-HT_2A_/5-HT_7_ antagonism while maintaining selectivity over other receptors, particularly the α_1_ adrenergic receptor. The investigation began by exploring various substituents on the N2 pyrazole nitrogen. As the size of the alkyl group increased from ethyl to isopropyl to *tert*-butyl, 5-HT_2A_ receptor activity improved consistently. However, 5-HT_7_ receptor potency showed a significant improvement from ethyl to isopropyl but then plateaued with further increase in size. This led to the conclusion that an isopropyl group at this position offered the optimal balance of activity at both 5-HT_7_ and 5-HT_2A_ receptors. With the N2 position optimized, attention turned to modifications of the aryl ring at the C3 position of the pyrazole. The SAR revealed that small lipophilic substituents were well-tolerated at this position. Specifically, methyl, chloro, and fluoro groups in the *para* position of the pendant aryl ring provided excellent potency at both 5-HT_2A_ and 5-HT_7_ receptors. However, attempts to increase the size or polarity of the substituent were less successful. The addition of a second chlorine atom resulted in a reduction of 5-HT_7_ potency, as did the introduction of a more polar nitrile group. Through this systematic exploration, compound **43** emerged as the most promising candidate. This compound, featuring an isopropyl group on the N2 pyrazole nitrogen and a *para*-fluoro substituent on the aryl ring, demonstrated the most desirable combination of 5-HT_2A_ and 5-HT_7_ potency. Moreover, **43** exhibited the best selectivity ratio over the α_1_ adrenergic receptor and a relatively low calculated log *P* (*c* log *P*) among the compounds tested, suggesting favorable drug-like properties.

*In vivo* studies further corroborated the therapeutic potential of compound **43**. In a rat model assessing 5-HT_7_ receptor activity, **43** demonstrated significant efficacy at very low doses, with an ED_50_ of 0.05 mg/kg for the inhibition of 5-CT-induced hypothermia. Maximal effect was observed at 0.3 mg/kg *po*, corresponding to a plasma concentration of approximately 27 ng/mL. In a mouse model evaluating activity at 5-HT_2A_ receptor, **43** effectively blocked DOI-induced head-twitches with an ED_50_ of 0.3 mg/kg *po*. These findings, combined with favorable ADME properties and high oral bioavailability across multiple species, led to the selection of compound **43** (JNJ-26528398) as a clinical candidate.

## 6 Conclusions

Despite decades of progress in receptor pharmacology and drug development, the treatment of CNS disorders remains a significant challenge. Traditional strategies targeting a single receptor or employing multitarget strategies have yielded some clinical benefit but are often limited by suboptimal efficacy, delayed therapeutic onset, and adverse side effects. The development of dually acting ligands that modulate two serotonergic sites or simultaneously target serotonergic receptors and the transporter offers a promising avenue to overcome these limitations. Furthermore, compared to the rapid progress in anticancer research, CNS drug discovery continues to face stagnation, partly due to the absence of clearly defined receptor signaling pathways and the limited predictive validity of existing preclinical models. Against this background, rational design of dually acting serotonergic agents exemplifies emerging strategies aimed at revitalizing neuropsychopharmacology.

From approved dually acting drugs as pimavanserin, 5-HT_2A_/5-HT_2C_ receptors inverse agonist, and ulotaront, which behaves as TAAR1/5-HT_1A_ receptor agonist to investigational compounds that target selected 5-HT receptor subtypes, the field continues to evolve. These include eltoprazine (a partial 5-HT_1A_/5-HT_1B_ receptors agonist), FPPQ (5-HT_3_/5-HT_6_ receptors antagonist), PZKKN-94 (5-HT_1B_ receptor agonist/5-HT_6_ receptor antagonist), and agents with combined 5-HT_4_ receptor agonistic/5-HT_6_ receptor antagonistic activity or TAAR1/5-HT_2C_ receptor agonistic activity (Figure 17).

**Figure 17:**
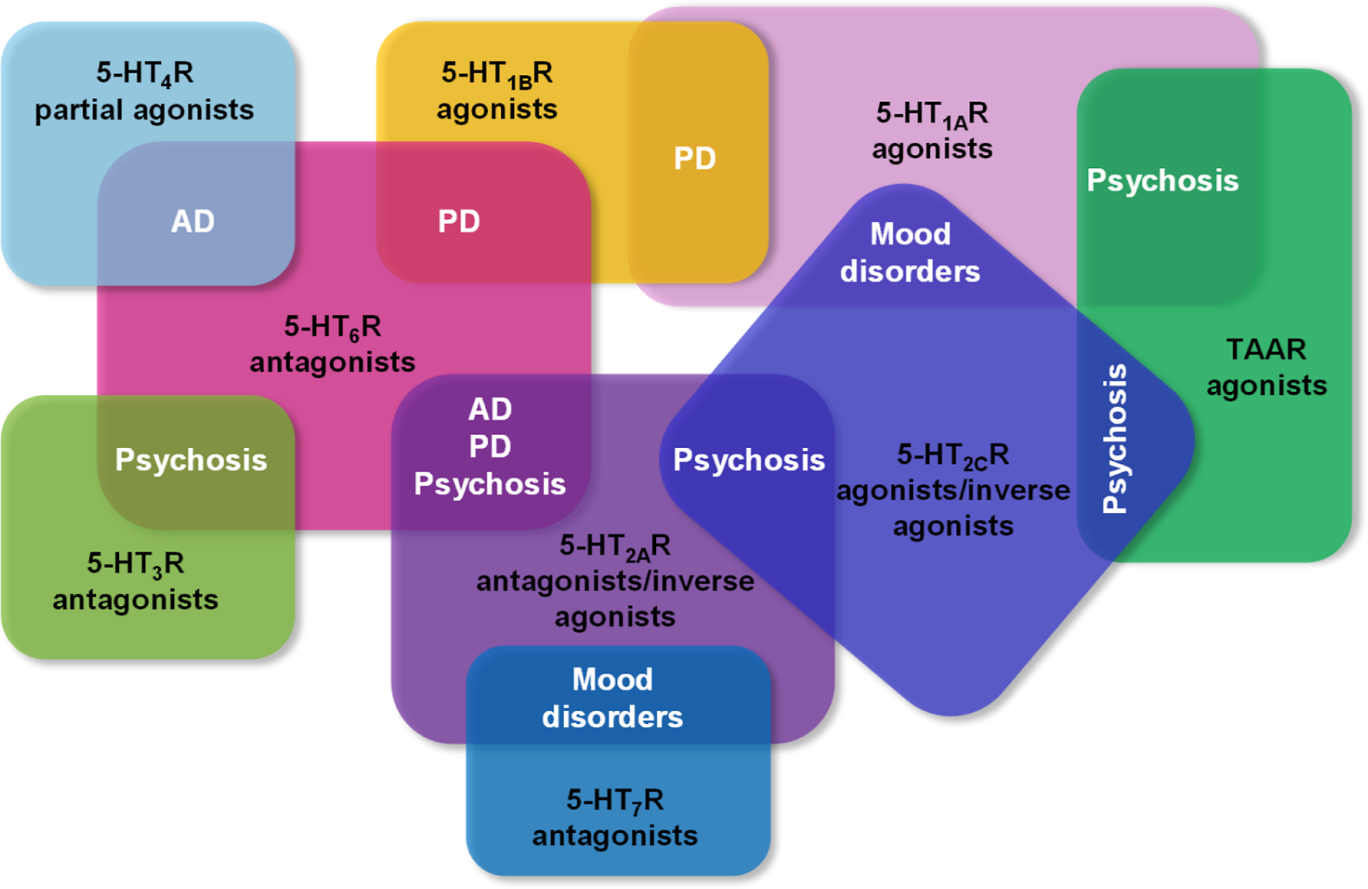
Schematic representation of the interplay between 5-HT receptor subtypes and TAAR across major CNS disorders. The overlapping regions depict shared receptor involvement in the potential treatment strategies among AD, PD, psychosis, and mood disorders.

Accumulating evidence consistently supports the hypothesis that balanced modulation of two complementary targets may enhance therapeutic efficacy, broaden symptom coverage, and overcome limitations inherent to one-target approaches (Table 1).

**Table 1:** Mechanisms of Serotonergic and Trace Amine Receptor Modulation: Effects and Likely Significance of Dual Activity in Neurodegenerative and Neuropsychiatric Disorders

mechanism	effects	likely significance of dual activity
5-HT_1A_ receptor agonism	improvement of motor symptoms and nonmotor symptoms of PD	purported antidyskinetic activity in PD, but progression of motor impairments in PD patients was observed
5-HT_1B_ receptor agonism	antidyskinetic effect	example compound: eltoprazine (*investigated in clinical trials*)
	antiparkinsonian-like effects in the rat models of PD	
5-HT_1A_ receptor partial agonism	anxiolytic activityantidepressant activity	antipsychotic and antidepressant effects in schizophrenia and major depression
TAAR agonism	antidepressant activity	example compound: ulotaront (*ongoing clinical development*)
	antipsychotic activity	
		procognitive activity
5-HT_1B_ receptor agonism	antidepressant effect	purported antiparkinsonian effects, attenuation of motor fluctuations (“on–off” phenomena) with no impact on l-DOPA efficacy, accompanied by antidepressant and procognitive effects
	antidyskinetic effect	example compound: PZKKN-94 (*requires further exploration in clinical trials*)
	antiparkinsonian activity	
5-HT_6_ receptor antagonism	antidepressant activity	
	antiparkinsonian effect	
		procognitive activity
5-HT_2A_ receptor inverse agonism at Gαi1 signaling pathway/5-HT_2C_ receptor inverse agonism	antipsychotic activity	favorable antipsychotic effect in PD patients
		example compound: pimavanserin (*approved*)
5-HT_2A_ receptor antagonism	antipsychotic activity	Purported enhanced procognitive activity was not confirmed in schizophrenic and AD patients
5-HT_6_ receptor antagonism	procognitive activity	example compound: idalopirdine (*investigated in clinical trials*)
5-HT_2A_ receptor antagonism	antipsychotic activity	purported antidepressant effect
5-HT_7_ receptor antagonism	antidepressant activity	example compound: JNJ-26528398 (*requires further exploration in clinical trials*)
	procognitive activity	
5-HT_2C_ receptor agonism	antipsychotic activity	potential effect on negative schizophrenic symptoms and procognitive activity
TAAR agonism	antipsychotic activity, antidepressant-like activity procognitive activity	example compound: compound **37** (*requires further exploration in clinical trials*)
5-HT_3_ receptor antagonism	improving negative symptoms of schizophrenia	purported effect on positive schizophrenic symptoms and procognitive activity
	procognitive activity	example compound: FPPQ (*requires further exploration clinical trials*)
5-HT_6_ receptor neutral antagonism in the Gs pathway	procognitive activity	
5-HT_4_ receptor agonism	disease modifying opportunities	potential symptomatic and disease-modifying effect in AD
	procognitive activity	example compound: compound **11** (*requires further exploration in clinical trials*)
5-HT_6_ receptor antagonism	procognitive activity	

Recent insights into GPCR pharmacology further expand the design space for such ligands. Emerging data highlights the therapeutic relevance of functional selectivity or biased agonism, which enables ligands to preferentially activate specific intracellular signaling cascades coupled to serotonin receptors. For instance, 5-HT_2A_ receptor agonists can be tailored to favor either G_q_ or β-arrestin pathways, potentially yielding antidepressant or antipsychotic effects, respectively.^83,104,140^ A converging body of evidence indicates that 5-HT_2A_ receptor agonists act as psychoplastogens, rapidly enhancing synaptogenesis.^141,142^ Intriguingly, intracellular location bias in 5-HT_2A_ receptor signaling has been highlighted as a therapeutic target. Conversely, biased 5-HT_1A_ agonists targeting different signaling pathways and exhibiting region-selective activity within the brain may help accelerate the onset of antidepressant responses.^143^ Similarly, the ability of ligands to stabilize distinct receptor conformations, as seen with 5-HT_6_ neutral antagonists versus inverse agonists,^42,144,145^ opens new avenues for fine-tuning pharmacological outcomes and cytoprotective effects in astrocytes.

Extending the mechanistic discussion to 5-HT_4_ and 5-HT_7_ receptors could further enrich our understanding of GPCR functional selectivity and expand opportunities for precision pharmacology. In the case of the 5-HT_4_ receptor, agonists engineered to preferentially activate ERK-dependent signaling over the canonical Gs pathway may offer rapid antidepressant-like effects, procognitive benefits, and neurotrophic effects while minimizing peripheral side effects.^6^ Additionally, developing β-arrestin-biased ligands targeting the 5-HT_7_ receptor presents a promising nonopioid therapeutic avenue for analgesia, leveraging pathway-specific modulation to dissociate antinociceptive efficacy from the liabilities associated with conventional opioid treatments.^146^

To validate these nuanced signaling hypotheses and advance the rational design of dually acting compounds, the field is exploring light-controlled pharmacology. This approach employs two primary strategies: photolabile compounds (caged ligands), which irreversibly release active agents upon irradiation, and photoswitchable probes, which reversibly toggle between isomeric states (e.g., *trans* vs *cis*) to dynamically modulate receptor activity. Leveraging the latter, recent examples such as TCB-2-Azo and Azo-5-HT^147^ serve as reversible optical switches, offering precise spatiotemporal control over *in situ* control of the 5-HT_2A_ receptor. These sophisticated probes enable the dynamic manipulation of functional selectivity, facilitating a targeted shift in signaling between G protein signaling and b-arrestin recruitment under specific wavelengths. This capacity is pivotal for deconvoluting the complex CNS mechanisms underlying therapeutic and adverse effects. Such mechanistic clarity is indispensable for the rational design of next-generation, biased dually acting compounds with enhanced therapeutic profiles. Advances in computational drug design—including pharmacophore mapping, machine learning, and AI-driven prediction—are accelerating the identification and optimization of such dually acting agents. These tools enhance our ability to characterize receptor–ligand interactions and predict dual-target activity with greater precision.^148–150^

Together, investigation into the functions of 5-HT receptor subtypes, receptor states, and their downstream signaling pathways remains a strong foundation for developing of dually acting agents with improved efficacy and safety profiles (Figure 18). This strategy holds significant promise for addressing the unmet needs and applicable across a broad spectrum of neuropsychiatric conditions. Collectively, these insights challenge conventional assumptions and guide future mechanism-driven CNS drug discovery.

**Figure 18:**
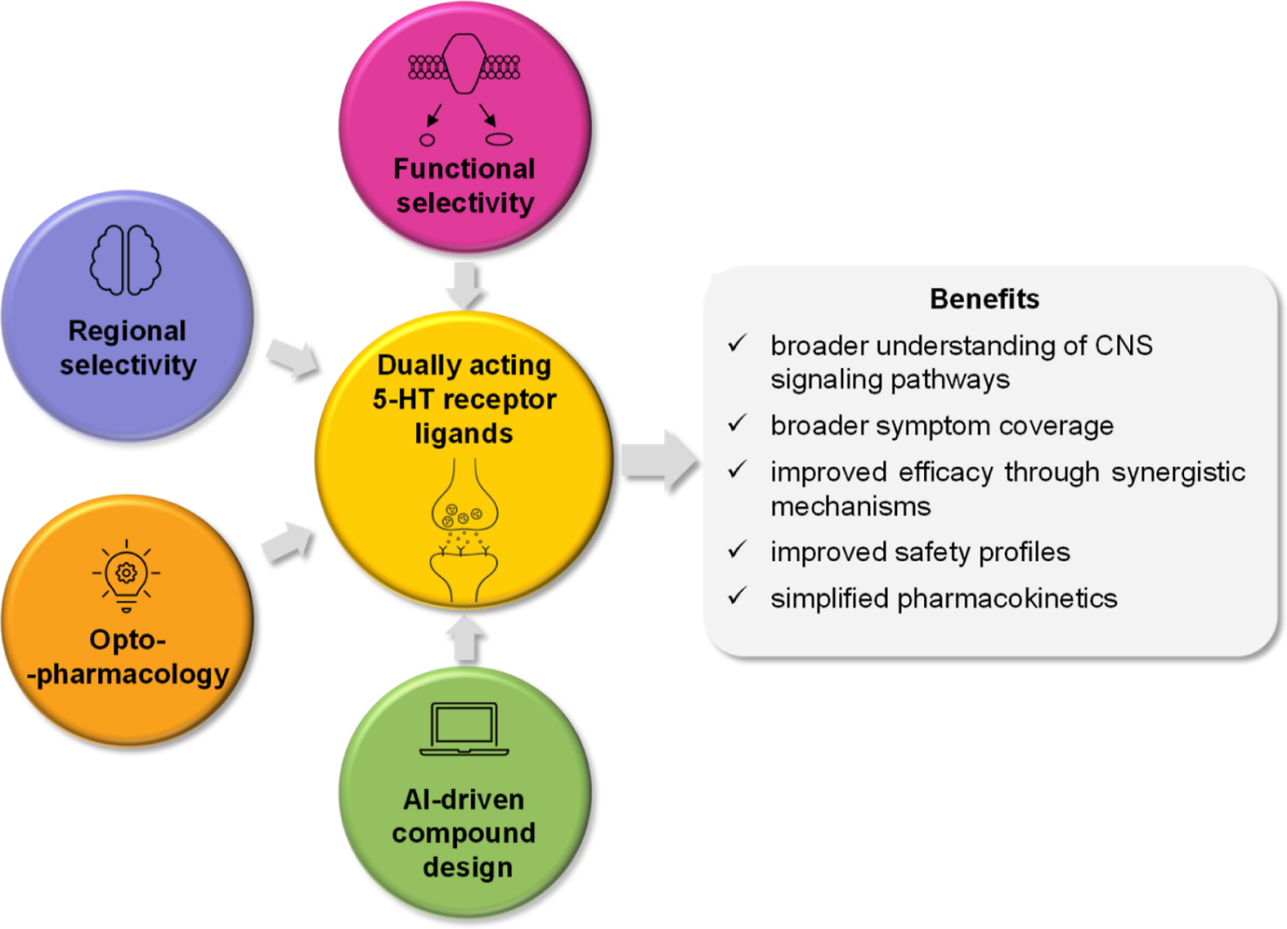
New directions in the development of dually acting 5-HT receptors ligands.

## Abbreviations:

6-OHDA: 6-hydroxydopamine
AD: Alzheimer’s disease
AI: artificial intelligence
ALS: amyotrophic lateral sclerosis
APP: amyloid precursor protein
Aβ: amyloid-β
BACS: Brief Assessment of Cognition in Schizophrenia
BBB: blood–brain barrier
BRET: Bioluminescence Resonance Energy Transfer
cAMP: cyclic adenosine monophosphate
CBD: cannabidiol
CGRP: calcitonin gene-related peptide
Cdk5: cyclin-dependent kinase type 5
CNS: central nervous system
CRH: corticotropin-releasing hormone
CSDS: chronic social defeat stress
D2: dopamine type-2
D4: dopamine type-4
DLB: dementia with Lewy bodies
DRN: dorsal raphe nucleus
DRP: dementia-related psychosis
EPO: European Patent Office
ERK: extracellular signal-regulated kinase
FDA: Food and Drug Administration
GPCRs: G protein-coupled receptors
HD: Huntington’s disease
HSDD: hypoactive sexual desire disorder
l-DOPA: levodopa
LIDs: levodopa-induced dyskinesias
mTOR: mechanistic target of rapamycin
MPTP: 1-methyl-4-phenyl-1,2,3,6-tetrahydropyridine
NET: norepinephrine transporter
NOR: novel object recognition
OCT3: organic cation transporter 3
PANSS: Positive and Negative Syndrome Scale
PCP: phencyclidine
PD: Parkinson’s disease
PDP: Parkinson’s disease psychosis
PET: positron emission tomography
PFC: prefrontal cortex
PMAT: plasma membrane monoamine transporter
PPI: prepulse inhibition
SAR: structure–activity relationship
SERT: serotonin transporter
SNRI: serotonin–norepinephrine reuptake inhibitors
SSRIs: selective serotonin reuptake inhibitors
TAAR: trace amine-associated receptor
VTA: ventral tegmental area
